# Perspectives on NO_*X*_ Emissions and Impacts from Ammonia Combustion Processes

**DOI:** 10.1021/acs.energyfuels.4c03381

**Published:** 2024-10-02

**Authors:** Syed Mashruk, Hao Shi, Luca Mazzotta, Cihat Emre Ustun, B. Aravind, Roberto Meloni, Ali Alnasif, Elena Boulet, Radoslaw Jankowski, Chunkan Yu, Mohammad Alnajideen, Amin Paykani, Ulrich Maas, Rafal Slefarski, Domenico Borello, Agustin Valera-Medina

**Affiliations:** † College of Physical Sciences and Engineering, Cardiff University, Cardiff, Wales CF24 3AA, U.K.; ‡ Reaktive Strömungen und Messtechnik (RSM), TU Darmstadt, 64287 Darmstadt, Germany; § Department of Astronautical, Electric and Energy Engineering, Sapienza University of Rome, Via Eudossiana 18, Rome 00184, Italy; ∥ Baker Hughes, Via F. Matteucci 2, Firenze 50127, Italy; ⊥ School of Engineering and Materials Science, Queen Mary University of London, London E1 4NS, U.K.; # Engineering Technical College of Al-Najaf, Al-Furat Al-Awsat Technical University, Najaf 31001, Iraq; ∇ Institute of Thermal Engineering, Poznan University of Technology, 60-965 Poznan, Poland; ○ Institute for Technical Thermodynamics, KIT—Karlsruhe Institute of Technology, 76131 Karlsruhe, Germany; ◆ Department of Mechanical and Aerospace Engineering, Sapienza University of Rome, Via Eudossiana 18, Rome 00184, Italy

## Abstract

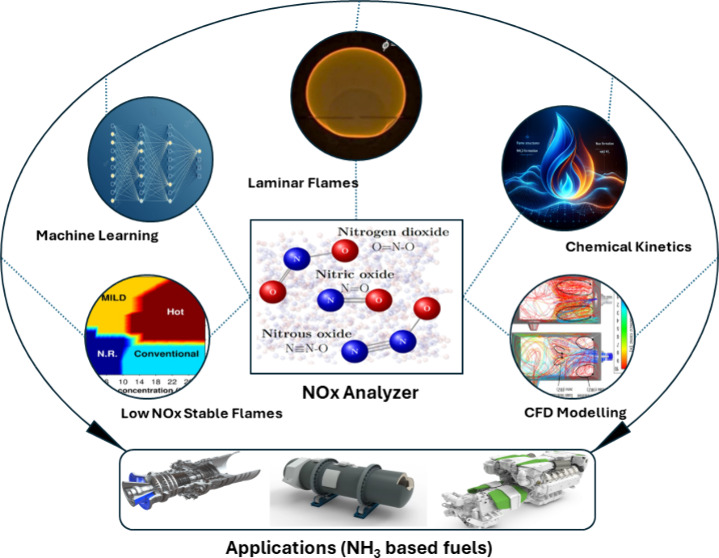

Climate change and global warming necessitate the shift toward low-emission, carbon-free fuels. Although hydrogen boasts zero carbon content and high performance, its utilization is impeded by the complexities and costs involved in liquefaction, preservation, and transportation. Ammonia has emerged as a viable alternative that offers potential as a renewable energy storage medium and supports the global economy’s decarbonization. With its broader applicability in large power output applications, decentralized energy sources, and industrial locations off the grid, ammonia is increasingly regarded as an essential fuel for the future. Although ammonia provides a sustainable solution for future low-carbon energy fields, its wide-scale adoption is limited by NO_*X*_ emissions and poor combustion performance under certain conditions. As research on ammonia combustion expands, recent findings reveal factors impacting the chemical reaction pathways of ammonia-based fuels, including the equivalence ratio, fuel mixture, pressure, and temperature. Investigations into ammonia combustion and NO_*X*_ emissions, at both laboratory and industrial scales, have identified NO_*X*_ production peaks at equivalence ratios of 0.8–0.9 for ammonia/hydrogen blends. The latest studies about the NO_*X*_ emissions of the ammonia flame at different conditions and their generating pathways are reviewed in this work. Effective reduction in NO production from ammonia-based flames can be achieved with richer blends, which generate more NH_*i*_ radicals. Other advanced NO_*X*_ mitigation techniques such as plasma-assisted combustion have been also explored. Further research is required to address these challenges, reduce emissions, and improve efficiencies of ammonia-based fuel blends. Finally, the extinction limit of ammonia turbulent flame, its influential factors, and different strategies to promote the ammonia flame stability were discussed. The present review contributes to disseminating the latest advancements in the field of ammonia combustion and highlights the importance of refining reaction mechanisms, computational models, and understanding fundamental phenomena for practical implications.

## 1 Introduction

The global demand for energy is expected to increase significantly in the coming decades, with the International Energy Agency (IEA) predicting a 3-fold increase in the next 10 years and a 5-fold increase by the middle of the century.^1^ This rise in energy demand, coupled with the escalating concentration of carbon dioxide in the atmosphere, presents a complex and critical challenge for humanity. Historically, the increase in energy requirements has led to a surge in emissions, resulting in climate changes and associated consequences.

According to the Intergovernmental Panel on Climate Change (IPCC), climate change is primarily caused by emissions such as carbon dioxide, which have already raised global temperatures by 0.8–1.2 °C. It is estimated that by 2050 the average increase will surpass 1.5 °C, and there are concerns that it may reach 2.0 °C, a threshold beyond which the impacts on climate and the environment could become irreversible.^2^ Therefore, the consequences of human activities and pollution will directly impact living standards and economic performance across regions.^3^

In response to these challenges, novel methods of energy generation have been conceived, such as wind, solar, and marine energies, which are being explored as sustainable alternatives. However, wind and solar energy systems face significant challenges. Their availability varies based on weather and seasonal conditions, making them intermittent energy sources. Moreover, large amounts of these energies are often stranded in remote locations that are economically impractical to connect directly to local grids. Marine energy, on the other hand, is hindered by high costs, challenging market environments, and the need for consistent energy production at reliable power outputs. These factors, along with political, social, and public perception considerations, can impede the widespread adoption of renewable energies that could reduce the dependence on fossil fuels and mitigate climate change emissions.

Among various methods of energy storage, chemical energy storage has been utilized for centuries, exemplified by our reliance on fossil fuels. Hence, the use of chemicals with low or no carbon content presents a unique solution for harnessing renewable, intermittent resources. Hydrogen storage is one such avenue, offering a chemical with zero carbon content and a high gravimetric energy density. However, hydrogen storage presents intrinsic challenges. Being the smallest molecule in the universe, hydrogen tends to permeate a wide range of materials, and its control in large-scale power generation systems, such as combustion-based systems, poses stability issues. Additionally, the explosive nature and fast-burning velocities of hydrogen make it difficult to handle compared with conventional sources. Moreover, the long-term storage costs of hydrogen can be economically unfeasible for many applications, as the molecule needs to be cooled to cryogenic conditions below 20 K (−253 °C).^4^ These limitations have spurred the exploration of other molecules, supporting the concept of a “hydrogen economy”.

Ammonia, a molecule with a history spanning over 180 years, offers a unique platform for hydrogen storage and the delivery of renewable energy. Compared to hydrogen, ammonia (NH_3_) boasts a higher volumetric energy density (4325 vs 1305 Wh/L), higher liquefaction temperature (293.8 vs 20 K), and lower storage pressure (8 vs 700 bar), making it a promising alternative with high reliability and low cost.^5^ Currently, the main method of ammonia production is the Haber–Bosch process, carried out in super-large-scale plants capable of producing between 2000 and 3000 tons/day, a capacity expected to increase substantially in the coming years.^6^ Due to its feasibility in production, preservation, and distribution, ammonia is considered a sustainable option to meet the energy requirements of future low-carbon economies.

While ammonia has gained considerable acceptance in the industrial sector for power, heat, and propulsion generation through combustion systems, there are still unresolved questions that require further research and development. These include the reduction of emissions such as NO and N_2_O, as well as unburned ammonia and carbon monoxide during cofiring processes. Therefore, gaining a better understanding of the mechanisms involved in the formation of NO_*X*_ products from ammonia is crucial for finding solutions to suppress these undesirable species.

This review addresses a critical gap in the existing literature by providing a comprehensive and timely analysis of NO_*X*_ emissions from ammonia combustion across various fuel blends and combustion applications, such as gas turbines, boilers, furnaces, and internal combustion engines (ICEs). While previous reviews, such as those by Tian et al.,^7^ Elbaz et al.,^8^ and Kobayashi et al.,^9^ have explored the fundamentals and specific aspects of ammonia combustion, this paper integrates the latest advancements in chemical kinetics mechanisms, reaction pathways of NO_*X*_ generation, and computational fluid dynamics (CFD) studies. This review also provides a holistic perspective on the challenges and potential solutions (e.g., MILD combustion) for reducing NO_*X*_ emissions in practical combustion systems.

Given the increasing interest in ammonia as a hydrogen carrier and its potential role in a hydrogen economy, understanding its combustion characteristics and environmental impacts is essential. This review fills a critical gap in the current literature by offering valuable insights for engineers and policymakers working on clean energy technologies. It identifies specific challenges and provides clear directions for future research, such as the need for improved chemical kinetics models and optimized combustion systems. By bridging the gap between fundamental combustion science and applied technology, this review contributes significantly to the advancement of the field.

## 2 NO_*X*_ Emissions in Ammonia Combustion

To improve the design of combustion systems with the aim of minimizing the environmental impact of NO_*X*_ emissions, it is essential to understand the factors contributing to their increase. This requires a thorough investigation of the chemical kinetics behind NO_*X*_ emissions, which should be continually updated based on experimental data. NO_*X*_ refers to various forms of nitrogen oxides produced during combustion, primarily nitric oxide (NO), nitrogen dioxide (NO_2_), and nitrous oxide (N_2_O). NO is a colorless gas that acts as a precursor to NO_2_ and plays a significant role in photochemical reactions that lead to smog formation.^10^ NO_2_ is particularly harmful as it can damage the respiratory system and, through reactions with water, oxygen, and other atmospheric chemicals, contribute to the formation of acid rain.^11^ This rain adversely affects lakes and forests, degrading the ecosystem. N_2_O, with its strong greenhouse effect, has a global warming potential 300 times greater than that of CO_2_, thus presenting a severe threat to the environment.^11,12^ Therefore, all forms of nitrogen oxides are major air pollutants that must be carefully managed in combustion processes, especially when NH_3_ (ammonia) is introduced as a fuel.

One of the main obstacles in the deployment of ammonia as a fuel is its proficiency in fuel NO_*X*_ production. Fuel NO_*X*_ is formed when nitrogen is chemically bonded to the fuel—which is the case for ammonia (NH_3_)—through the production of intermediate products, such as CN, HCN, HNO, and NH_*i*_, and further oxidation. Considerable amounts of thermal NO_*X*_ can also be generated with high enough flame temperatures.^13^ Nitrogen oxides present a significant risk to both health and the environment. These emissions can affect drinking water distribution,^14^ cause eutrophication,^15^ and aggravate lung diseases if inhaled.^16^ Nitrous oxide (N_2_O), another prospective product of ammonia combustion at certain operating conditions, has 273 times the 20-year global warming potential (GWP_20_) of CO_2_.^17^ Recent studies of ammonia/hydrogen blends have reported 240 ppm N_2_O emissions to have a global warming impact approximately equal to that of CO_2_ emitted from pure methane flame operating at dry low NO_*X*_ (DLN) scenarios.^18^

### 2.1 Initial Work

The initial explorations of using ammonia as a potential candidate for combustion for power generation emerged in the 1960s. Newhall and Starkman^19^ pioneered a theoretical analysis of ammonia as a turbine fuel, considering the thermodynamic processes involved. They predicted that ammonia could yield a power output up to 10% greater than hydrocarbon fuels under the same conditions, with thermal efficiencies potentially 10% higher. However, they also noted that specific fuel consumption for ammonia would exceed that of hydrocarbons by 2.5–3 times due to the presence of inert nitrogen. Pratt^20^ focused on the performance of ammonia-fired gas turbine combustors, both theoretically and experimentally. The authors confirmed that the chemical reaction between ammonia and air was relatively slow, necessitating adjustments in combustor design to accommodate for less efficient mixing at reduced air flow rates. Pratt suggested that smaller fuel nozzle orifices or multiple combustors in parallel could be potential solutions to improve the combustion efficiency. Verkamp et al.^21^ conducted extensive experimental studies to determine the ignition energy, quenching distance, flame stability limits, and overall performance of ammonia–air mixtures in gas turbine burners. They found that ammonia required a minimum ignition energy of 8 mJ, significantly higher than propane’s less than 0.5 mJ. The quenching distance for ammonia–air was also larger than that for propane–air, indicating a less stable combustion process. The study concluded that neat ammonia could not be directly used as a substitute for hydrocarbons in conventional gas turbines without modifications to the ignition system and combustion chamber design.

From 1964 to 1966, the U.S. Army in collaboration with the Solar Division of International Harvester Co., provided valuable insights into the characteristics of ammonia as a fuel and the engineering innovations required for its effective utilization in gas turbine engines.^22^ The research involved a two-phase approach to address the complexities of ammonia combustion. Phase I focused on a broad range of tests and analytical studies to determine the feasibility of using ammonia in gas turbines. The phases included combustion system development, control and accessory adaptations, and material compatibility assessments. Phase II centered on modifying a 250 hp Solar T-350 gas turbine engine to operate on ammonia fuel with minimal changeover effort. The engine was equipped with two types of ammonia combustion systems: a vapor combustor and an oxidation-catalyst-aided combustor. Rigorous testing was conducted to ensure satisfactory engine performance and operation with both systems. The results indicated that while ammonia combustion is more challenging than hydrocarbon fuel combustion, it can be effectively managed with the right engineering solutions. The use of catalytic aids showed particular promise, offering an improved combustion efficiency and reduced combustor volume. The study concluded that ammonia can be a satisfactory substitute for hydrocarbon fuels in simple-cycle gas turbine engines. While the vapor combustor offered an adequate solution for certain applications, the catalytic combustion system showed potential for further development, particularly in enhancing response characteristics and reliability.

Moreover, the U.S. Army Aviation Materiel Laboratories assessed the feasibility of ammonia-fueled gas turbine engines for use in army aircraft,^23^ considering the Nuclear Powered Energy Depot Concept. The research evaluated the design and characteristics of high- and low-pressure tanks for storing ammonia, considering its low boiling point and the need for pressurized or refrigerated storage. The use of refrigerated tanks for long-term storage and the potential for gelation of ammonia to reduce the vapor pressure are also discussed. The study encompassed performance assessments in both rotary-wing (UH-1D helicopter) and fixed-wing (CV-7A aircraft) configurations and found that while ammonia could increase the maximum power output by approximately 15%, its lower heating value resulted in higher specific fuel consumption (SFC). The use of regeneration was considered crucial for improving the SFC of ammonia-fueled engines. This study also compared the mission radius, payload capacity, and productivity of these aircraft when using ammonia versus hydrocarbon fuels. It was concluded that ammonia-fueled aircraft exhibited significantly lower productivity and reduced mission radius capabilities. The researchers concluded that ammonia as a gas turbine fuel resulted in considerably lower aircraft productivity compared to hydrocarbon fuels. The specific fuel consumption for ammonia-fueled engines was found to be 2.20–2.6 times higher than that for hydrocarbon-fueled engines, while the maximum power output was only moderately higher. It was suggested that alternative technologies, such as variable compressor and turbine geometry, high-temperature turbines, and regeneration, should be explored to reduce hydrocarbon fuel logistics.

In the realm of aerospace, ammonia-based technology marked its foray into propulsion systems, exemplified by the development of the X-15 aircraft at the National Advisory Committee for Aeronautics (NACA) Langley Aeronautical Laboratory, which has since evolved into the NASA Langley Research Centre.^24^ This vehicle was propelled by the XLR-99 engine, a throttleable powerhouse capable of generating up to 250 500 N of thrust. The engine’s propulsion system was uniquely designed to utilize a combination of liquid ammonia and liquid oxygen (LOX), which together provided the necessary energy for its operation.

The selection of liquid ammonia as a fuel component was motivated by its stability and favorable volumetric energy density, which facilitated storage under the stringent conditions demanded by high-performance aerospace applications. Additionally, the cooling effects derived from the use of liquid propellants significantly enhanced the engine’s operational efficiency. The X-15 was launched from a B-52 research aircraft, which itself was in flight at velocities approaching 800 km/h.

Despite these advancements, certain sources have^25^ indicated that the program’s discontinuation was precipitated by an incomplete appreciation for the ammonia system’s potential and by the pursuit of fuels with higher gravimetric energy density. However, the X-15’s legacy endures, as it achieved speeds of up to 6.7 times the speed of sound, a record that stood unmatched until the era of space shuttle travel.^24^ The X-15’s remarkable performance underscores the potential of ammonia as a fuel in aerospace applications, even as it highlights the challenges that must be surmounted to fully realize such technologies.

Recent advancements in ammonia combustion technology for power and propulsion have garnered significant attention. However, a primary challenge hindering its widespread adoption is the substantial NO_*X*_ emissions associated with the combustion process. Factors such as operating load, ambient conditions, and fuel mixture composition significantly influence the NO_*X*_ formation. To address this environmental concern, stringent regulations have been implemented, mandating a reduction in NO_*X*_ emissions from 200 ppm to as low as 25 ppm or even 9 ppm.^26^

While NO_*X*_ emissions generally increase with engine power, reaching their peak at the rated output, technologies like SCO Duiker’s have demonstrated the feasibility of combusting ammonia-rich streams (up to 100 vol %) in commercial applications, including furnaces and boilers in the processing and oil/gas extraction sectors. Mitsubishi Power’s ongoing development of a 100% ammonia-fed boiler further underscores the potential for stable ammonia combustion and NO_*X*_ suppression. It is imperative to note that, during ammonia combustion in conventional dry low NO_*X*_ (DLN) combustion systems, nitrogen from the ammonia fuel is more prone to conversion into NO_*X*_ compared to methane or hydrogen flames at similar temperatures. This can result in NO_*X*_ emissions exceeding 1000 ppm, even with blends of ammonia and natural gas.^27^ Consequently, extensive research is required to develop effective NO_*X*_ suppression strategies for ammonia combustion across various operating conditions.

Beyond thermal NO_*X*_ formation, the direct formation of N_2_O and NO_*X*_ from ammonia fuel is another critical factor. While higher ammonia fuel fractions can lead to lower thermal NO_*X*_ emissions, they may also increase direct NO and N_2_O formation. Pochet et al.^28^ observed a temperature-dependent trade-off between NO and N_2_O, where conditions favoring the reduction of one species often promoted the formation of the other. Furthermore, fuel-rich operation can result in residual unburned hydrogen due to incomplete ammonia combustion. Hayakawa et al.^29^ identified a crossover air–fuel ratio of approximately 1.05 where unburned ammonia, hydrogen, and residual NO were minimized. Fuel-richer operation tends to produce higher levels of NH_3_ and H_2_ in the exhaust, while stoichiometric or fuel-leaner conditions can lead to NO_*X*_ emissions exceeding 1000 ppm. However, the potential for fuel-rich operation in ammonia engines, which is not feasible with hydrocarbon fuels due to soot and CO formation, presents an opportunity for optimizing emissions. To fully understand the trade-offs between thermal- and fuel-borne NO_*X*_ and N_2_O formation across a wide range of operating conditions, further research is essential. By addressing these challenges, the adoption of ammonia as a sustainable fuel source can be accelerated while minimizing its environmental impact.

### 2.2 Ammonia Laminar Flames

Ammonia, as a carbon-free fuel, holds promise for reducing greenhouse gas emissions in combustion processes. However, the formation of NO_*X*_ during ammonia combustion poses a significant environmental challenge. Laminar flames, characterized by their stable and controlled nature, offer an ideal platform for dissecting the fundamental aspects of combustion and the intricate mechanisms of NO_*X*_ formation. This section endeavors to synthesize recent findings on NO_*X*_ emissions in ammonia laminar flames, focusing on the underlying chemical kinetics, the influence of various parameters on emission formation, and potential strategies for emission reduction.

The formation of NO_*X*_ in ammonia flames is predominantly governed by two mechanisms: the thermal (Zeldovich) mechanism and the fuel nitrogen mechanism.^30^ The thermal mechanism, which becomes dominant at temperatures exceeding 1800 K, involves the oxidation of atmospheric nitrogen to NO. Conversely, the fuel nitrogen mechanism is concerned with the conversion of nitrogen species bound within the fuel, such as NH_3_, to NO through a series of intermediate reactions. Lindstedt et al.^31^ provided pioneering insights into the chemical kinetics of ammonia oxidation, elucidating the pivotal role of HNO as an intermediate in NO formation. The study underscored the dependency of HNO conversion pathways on flame conditions, highlighting the significance of the NH with OH reaction in pure ammonia flames and the increasing importance of the Zeldovich mechanism in ammonia-doped hydrogen flames with increasing fuel concentrations. Sullivan et al.^32^ further explored the conversion dynamics of NH_3_ to NO_*X*_, finding that increased NH_3_ seeding leads to a higher conversion to N_2_ rather than NO, particularly in nonpremixed methane–air flames, emphasizing the complex interplay between fuel-nitrogen chemistry and NO_*X*_ formation.

The presence of hydrogen in ammonia flames introduces significant alteration in NO_*X*_ formation pathways, potentially enhancing NO production rates. Lee et al.^33^ demonstrated that the presence of hydrogen in ammonia flames could lead to higher NO production rates, an effect moderated under fuel-rich conditions, suggesting an optimal operational window for emission minimization. Hayakawa et al.^34^ further explored the product gas characteristics in ammonia/hydrogen/air premixed laminar flames, revealing a substantial increase in NO emissions at lean conditions, particularly when compared to pure ammonia/air flames. The study also identified a critical trade-off between NO and unburnt ammonia under slightly rich conditions and a rapid increase in N_2_O mole fraction around an equivalence ratio of 0.6, underscoring the importance of equivalence ratio control in reducing N_2_O emissions.

Wang et al.^35^ studied the laminar burning velocities (LBVs) of NH_3_/H_2_ blends at elevated pressures, and found that LBVs decrease with increasing ammonia mole fraction in the fuel mixture, while the addition of H_2_ significantly increases flame speed. The study concluded that ammonia blended with H_2_ can enhance combustion characteristics and promote NO formation by enriching the H and OH radical pools and increasing the flame temperature. Mei et al.^36^ employed a partial fuel cracking strategy to decompose NH_3_ into H_2_ and N_2_, leveraging the high reactivity of H_2_ to improve combustion characteristics. The LBVs of partially cracked NH_3_/air mixtures increased with the cracking ratio and peaked at equivalence ratios of around 1.1. The highest LBV measured was 38.1 cm/s at 1 atm, which is close to that of methane/air mixtures at the same pressure, indicating the effectiveness of the cracking strategy. Zheng et al.^37^ investigated the impact of radiation reabsorption on the laminar flame speed and NO emissions of NH_3_/H_2_/air mixtures under stoichiometric conditions with varying hydrogen ratios. Radiation reabsorption significantly influenced flame speed with a maximum enhancement of 17% observed at the lowest hydrogen ratio (η = 0.2). The effect decreased as the hydrogen ratio increased. Radiation reabsorption promoted NO generation and emission, with the effect being more pronounced at lower hydrogen ratios. The promotion effect decreased monotonically with an increasing hydrogen proportion.

**Figure 1:**
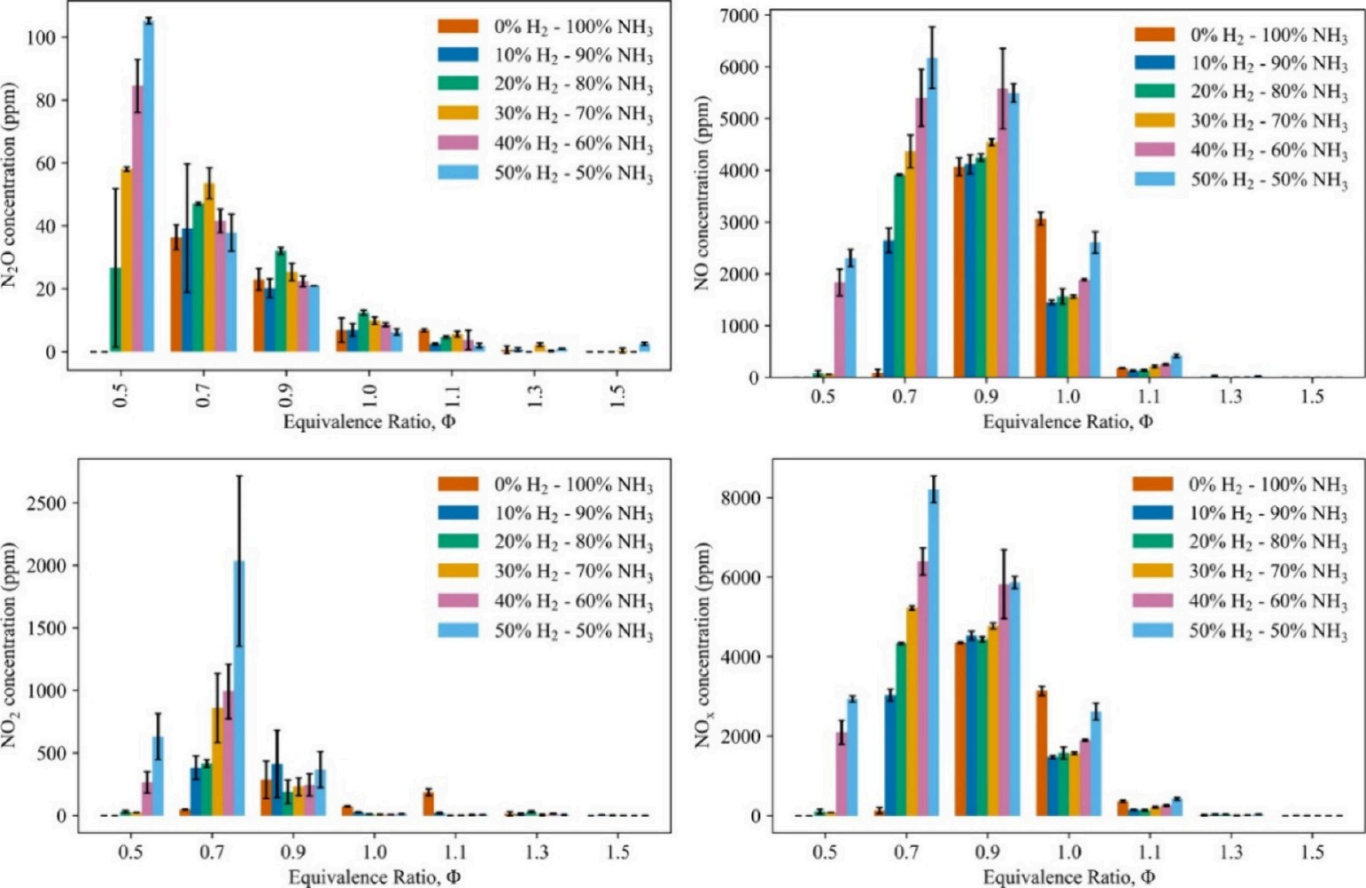
Recorded NO, NO_2_, N_2_O, and combined NO_*X*_ emissions. Three trial averages and associated standard deviations for N_2_O, NO, NO_2_, and combined NO_*X*_, from left to right and from top to bottom, respectively. Reprinted with permission from ref (38). Copyright 2024 Elsevier.

Nawaz et al.^38^ further delved into the combustion characteristics and NO_*X*_ emissions of ammonia–hydrogen blends in spherically expanding laminar flames. The hydrogen concentration in the fuel blends was varied from 0 to 50%, and the equivalence ratios were adjusted from 0.5 to 1.5. The study found that NO_*X*_ emissions peak at lean conditions and decrease significantly for rich mixtures, with hydrogen addition tending to increase NO_*X*_ emissions, particularly at lean equivalence ratios. Figure 1 illustrates the average concentrations of NO, NO_2_, and N_2_O emissions across different mixture compositions and equivalence ratios. The figure highlights the trend of NO_*X*_ emissions increasing with hydrogen fractions in lean mixtures.

Alnasif et al.^39^ evaluated the performance of various kinetic reaction mechanisms in predicting N_2_O mole fractions in NH_3_/H_2_ blends especially with the 70/30 vol % mixing ratio. The NH + NO ⇌ N_2_O + H reaction was identified as being dominant in N_2_O formation across all studied conditions. The consumption of N_2_O was primarily through the reactions N_2_O + H ⇌ N_2_ + OH and N_2_O (+M) ⇌ N_2_ + O (+M). The study concluded that while the Klippenstein (2018) model generally predicts N_2_O mole fractions accurately, its performance deteriorates under very lean conditions. The chemical reaction NH + NO ⇌ N_2_O + H significantly contributes to the formation of N_2_O under all of the tested conditions. The reduction of N_2_O is primarily controlled by specific reactions involving H and NO.

Ariemma et al.^40^ and Kovaleva et al.^41^ explored the nonlinear dependency of NO_*X*_ emissions on the ammonia ratio in NH_3_/CH_4_/air laminar flames and emphasized the role of methane in boosting OH radical production, thereby increasing NO_*X*_ production through ammonia oxidation. For lean conditions, many reactions involving NH and NH_2_ species with O, H, NO, and O_2_ had a high sensitivity for NO without strongly affecting the laminar burning velocity. The latter study also reported HCN emissions with changing ammonia content. HCN was experimentally measured and found to be a significant pollutant at rich conditions (ϕ = 1.20–1.35; *E*_NH_3__ = 0.6–0.1), similar to ammonia emissions. Okafor et al.^42^ investigated the laminar burning velocity of premixed NH_3_/CH_4_/air mixtures, focusing on the influence of ammonia concentration on flame characteristics. A new detailed chemical kinetics model was developed that accurately predicted laminar burning velocities and NO emissions. The velocity decreased with increasing ammonia concentration with the highest velocity measured at 38.1 cm/s for methane–air mixtures at 1 atm, close to values reported in the literature. The burned gas Markstein length increased with the equivalence ratio and ammonia concentration, indicating increased flame stretch sensitivity. Wang et al.^43^ measured the laminar burning velocities of NH_3_/CH_4_/air mixtures at elevated pressures and developed a kinetic mechanism that accurately models the combustion characteristics of these mixtures. The velocity decreased with an increasing ammonia concentration, with the highest velocities measured for methane–air mixtures. The study provided comprehensive data on the laminar burning velocities of NH_3_/CH_4_/air mixtures at elevated pressures, which are critical for the development of gas turbine combustors for these fuels. The CEU-NH_3_-Mech 1.1 mechanism showed improved accuracy in predicting combustion characteristics, especially under rich conditions and at elevated pressures.

The impact of oxygen-enriched conditions on the formation of NO_*X*_ in ammonia laminar flames has been a subject of recent investigations. Woo et al.^44^ reported a nonmonotonic trend in NO_*X*_ emissions with varying oxygen ratios in ammonia-doped methane flames, with maximum emissions observed at an oxygen ratio of 0.7. Mei et al.^45^ complemented this with an experimental and kinetic modeling study under oxygen enrichment and elevated pressure, identifying the interplay between oxygen content, equivalence ratio, and initial pressure on NO_*X*_ emissions. Moreover, numerous studies have investigated the combustion characteristics and emissions of DME–ammonia blends, which are a type of oxygenated fuel. Meng et al.^46^ developed a detailed chemical reaction mechanism for the dual-fuel combustion of NH_3_/DME, which accurately predicted combustion behaviors in both laminar burning and ignition models, including combustion characteristics and emissions, particularly the formation and reduction of NO. The study found that adding up to 50% DME to ammonia promotes NO formation, leading to higher NO production. However, a blend with 75% NH_3_ and 25% DME (D25) generates more NH and NH_2_ radicals, promoting the NO reduction reaction and resulting in approximately 9% less NO at an equivalence ratio of 0.7. CH_3_ radicals from DME can slightly increase NO production by promoting the conversion of HNO and NO_2_ to NO. Yu et al.^47^ investigated the emission characteristics, particularly NO_*X*_, CO, and unburned NH_3_, of NH_3_/DME/air premixed flames. They found that lean conditions and stoichiometric conditions favored NO formation, while rich conditions negatively impacted NO formation. Increasing the NH_3_ fraction from 50 to 90% in the fuel mixture effectively decreased NO emissions. NO_2_ emissions followed a trend similar to that of NO but were approximately an order of magnitude lower in concentration. Rich conditions are more suitable for controlling NO emissions in NH_3_/DME co-combustion. The study suggested that single-stage combustion may not be optimal for pollutant control in NH_3_/DME co-combustion, and staged combustion strategies or NO_*X*_ post-treatment devices may be necessary. Alekseev et al.^48^ investigated NO formation in NH_3_/DME fuel mixtures and concluded that NO formation decreases with an increasing equivalence ratio (ϕ) and peaks at a certain NH_3_ percentage in the fuel mixture, after which it decreases with further increases in NH_3_ content. The study reported that, for the range of initial mixture parameters studied, NO formation in NH_3_/DME flames is primarily determined by the NH_3_ submechanism, with the hydrocarbon component influencing radical concentrations and heat release.

For various NH_3_-containing mixtures (NH_3_/air, NH_3_/H_2_/air, NH_3_/CO/air, NH_3_/CH_4_/air, etc.), detailed kinetic models have been developed to predict the laminar flame speed and NO_*X*_ emissions, with validation against experimental data. The updated NH_3_ chemistry, particularly the NNH chemistry, was found to be crucial for capturing the temperature dependence of NH_3_/air flames and the influence of H_2_, CO, and CH_4_ on NO_*X*_ emissions.^49^ Ramos et al.^50^ investigated different ammonia/methane blends (*X*_NH_3__ = 0–0.7) at three equivalence ratios (0.8, 0.9, and 1.0) in a laboratory-scale laminar flame burner. The experimental results indicated an initial rise in NO_*X*_ emissions as the ammonia content in the fuel mixture was raised to 50% with a decreasing trend afterward. This trend is consistent across the equivalence ratios studied (0.8, 0.9, and 1). NO_*X*_ emissions decrease as the equivalence ratio moves toward fuel-lean conditions, indicating that the combustion process becomes cleaner with less fuel relative to oxygen. The rate of production analysis indicates that the HNO pathway is crucial for NO formation, and its consumption is mainly driven by reactions with N, NH, and NH_2_ radicals. The formation and consumption of NO are highly sensitive to the H_2_/O_2_ chemistry, which determines the concentrations of the O, OH, and H radicals. Sensitivity analysis revealed that reactions involved in the H_2_/O_2_ submechanism, which regulates the amount of O, H, and OH radicals as well as temperatures, most affect the process of NO formation and consumption. In contrast to NO_*X*_, CO and NH_3_ emissions were found to be quite low, indicating near-complete combustion of CH_4_ and NH_3_.

**Figure 2:**
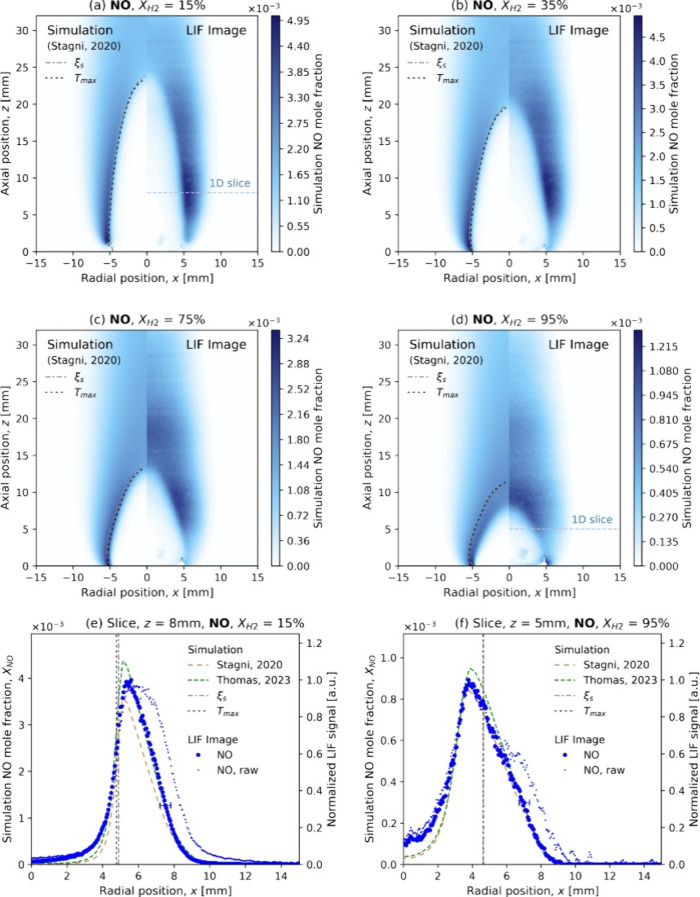
NO LIF images compared to simulation predictions for fuel hydrogen fractions of (a) 15, (b) 35, (c) 75, and (d) 95%. The species concentration profile for a 1D horizontal slice is also shown for (e) *X*_H_2__ = 15% (at the height indicated in (a)) and (f) *X*_H_2__) = 95% (at the height indicated in (d)). Reprinted with permission from ref (51). Copyright 2024 Elsevier.

Recently, Thomas et al.^51^ investigated the structure and nitrogen oxide (NO_*X*_) formation in laminar diffusion flames of ammonia–hydrogen fuel blends with air. The experimental approach involved blending ammonia fuel with hydrogen to enhance fuel reactivity, covering a fuel composition range of 15–100% hydrogen by mole fraction. The flame structure was visualized using planar laser-induced fluorescence (PLIF) for NO, NH, and OH radicals as well as OH* by filtered chemiluminescence. Numerical models were employed to predict species concentration profiles and were compared with experimental data. The study found that NO_*X*_ emissions, including NO, NO_2_, and N_2_O, decreased with increasing hydrogen content in the fuel blend. The authors also presented a detailed analysis of the reaction pathways contributing to NO creation and destruction, highlighting the importance of key reactions, such as HNO formation and the role of NH radicals. For high fuel hydrogen content, the measured NO profile shifted inward toward the fuel outlet, away from the peak temperature contour (see Figure 2), indicating a change in the flame structure and NO_*X*_ formation mechanisms.

The choice of fuel blend and operating conditions significantly impacts NO_*X*_ emissions in laminar ammonia flames. Hydrogen addition generally increases NO_*X*_ emissions, especially under lean conditions, due to increased flame speed and radical pool enrichment, with radiation reabsorption further promoting NO generation. In contrast, NH_3_/CH_4_ blends initially increase NO_*X*_ emissions with rising ammonia content up to 50%, followed by a decrease, with lean conditions favoring lower NO_*X*_ emissions. The NH_3_/DME blends show higher NO production up to 50% DME, but a blend with 75% NH_3_ and 25% DME reduces NO emissions under rich conditions due to increased NH and NH_2_ radicals. Oxygen enrichment can enhance NO_*X*_ emissions, but the effect can be complex and influenced by other factors. Optimizing fuel blends, operating conditions, and combustion strategies is crucial for minimizing NO_*X*_ emissions in ammonia-based combustion systems. For instance, while hydrogen addition can increase NO_*X*_ emissions, operating under fuel-rich conditions can mitigate this effect. Similarly, in NH_3_/DME blends, increasing the ammonia concentration can effectively reduce the NO_*X*_ emissions. The impact of oxygen enrichment on NO_*X*_ emissions is more nuanced, with nonmonotonic trends observed in some cases. To minimize NO_*X*_ emissions, a careful balance must be struck among fuel selection, operating conditions, and combustion strategies.

**Figure 3:**
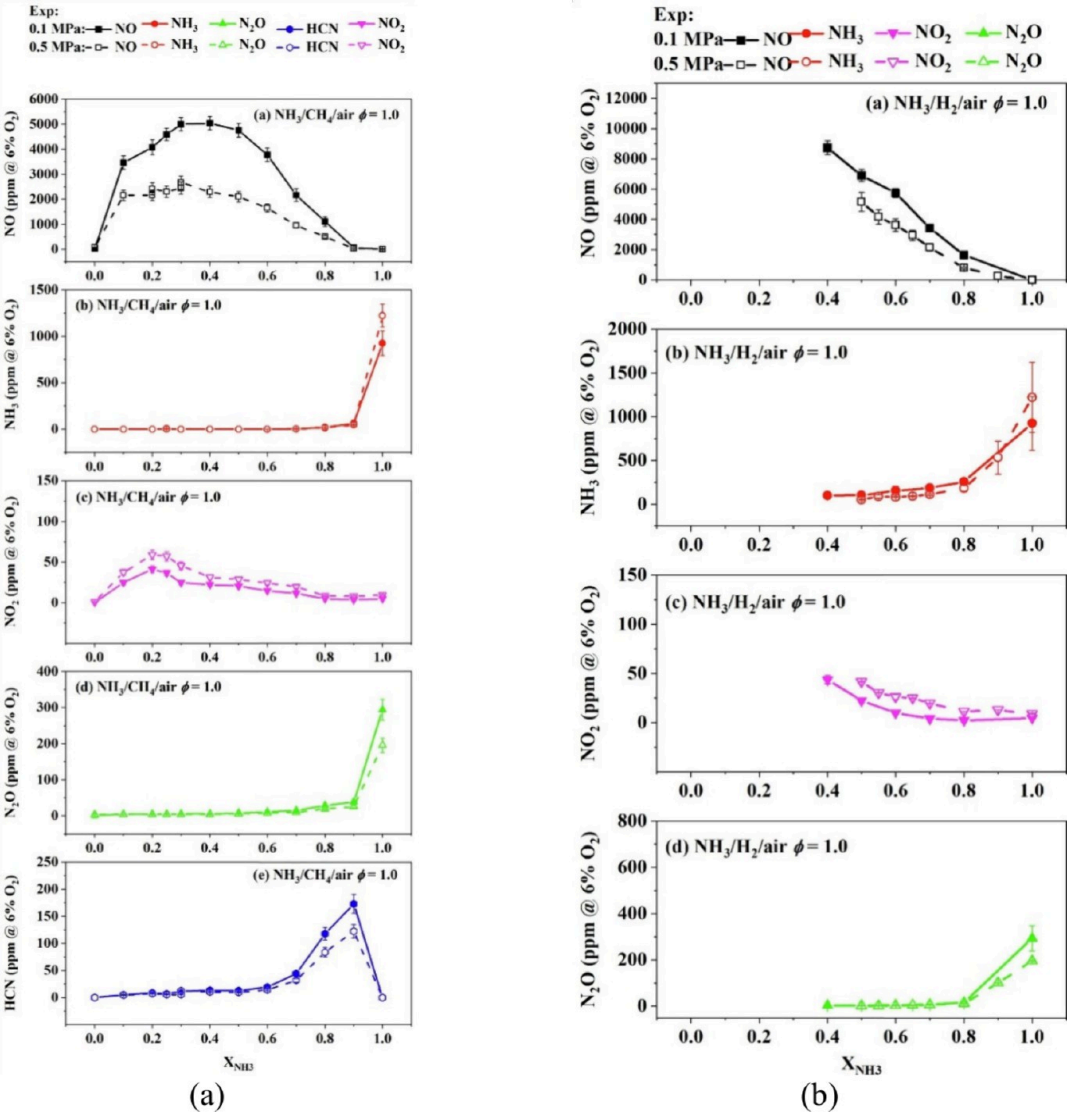
Measured and simulated NO, NH_3_, N_2_O, NO_2_, and HCN concentrations for the stoichiometric (a) NH_3_/CH_4_/air flame and (b) NH_3_/H_2_/air flame as a function of *X*_NH_3__ at 0.1 and 0.5 MPa (solid symbols, 0.1 MPa; open symbols, 0.5 MPa). Reprinted with permission from ref (52). Copyright 2024 Elsevier.

Moreover, Wang et al.^52^ presented a comprehensive study on the impact of elevated pressure and strain rate on NO_*X*_ emissions in laminar premixed flames of ammonia-enriched fuels. The research investigates the potential of combining elevated pressure and strain rate to reduce NO_*X*_ emissions in laminar premixed flames of NH_3_/CH_4_/air and NH_3_/H_2_/air. Utilizing a high-pressure heat flux burner and CHEMKIN software, the study measured and simulated NO_*X*_ emissions, including NO, NO_2_, N_2_O, NH_3_, and HCN. Key findings indicate that increasing pressure generally decreases NO_*X*_ emissions, with the exception of NO_2_, which sees an increase, as shown in Figure 3. Medium ammonia conditions are optimal for reducing the level of NO emissions through pressure enhancement. High strain rates were found to reduce NO emissions in stoichiometric and rich conditions but increased NO emissions in lean conditions before the NO peak equivalence ratio. The research concludes that the combination of elevated pressure and strain rate can significantly reduce NO_*X*_ emissions while ensuring complete ammonia oxidation, particularly for medium ammonia content at equivalence ratios between 0.9 and 1.1. This finding is instrumental for the design of practical high-pressure burners that utilize ammonia blend flames to minimize the NO_*X*_ emissions.

Ammonia laminar flames offer insights into the complex interplay among fuel chemistry, flame dynamics, and pollutant formation. This section provides a comprehensive understanding of the NO_*X*_ emission characteristics of ammonia laminar flames under various conditions. The synthesis of experimental and computational studies has been instrumental in identifying key factors influencing NO_*X*_ formation and in developing predictive models. While significant strides have been made, the need for further research to refine emission reduction strategies remains. The integration of experimental data with computational models provides a robust framework for predicting and mitigating NO_*X*_ emissions in ammonia combustion systems, crucial for the design of efficient and environmentally friendly combustors.

### 2.3 Ammonia Swirl Flames

#### 2.3.1 Pure Ammonia

Although the utilization of ammonia as a fuel offers a pathway to sustainable energy by reducing greenhouse gas emissions, the challenge of controlling NO_*X*_ emissions in these processes is nontrivial and requires a deep understanding of the underlying phenomena. This section reviews recent findings on the swirl flame of pure ammonia and its implications for NO_*X*_ emissions, drawing from various studies that have contributed to the understanding of the underlying chemical kinetics and combustion dynamics.

The formation of NO_*X*_ in ammonia swirl flames is a complex process influenced by various factors, including chemical kinetics and combustion dynamics. Klippenstein et al.^53^ have explored the role of the NNH radical of NO_*X*_ formation in ammonia/air flames. Recognized as a key intermediate in thermal De-NO_*X*_ processes, NNH is pivotal in the selective noncatalytic reduction of NO (NNH + NO = N_2_ + HNO), where ammonia acts as the reducing agent. This study investigated the potential energy surfaces of reactions involving NNH, revealing the impact of these reactions on NO formation (N_2_ + H = NNH, NNH + O = NH + NO, NH + O_*X*_ = NO + ...) and reduction. The updated chemical kinetics model presented in this research has been instrumental in enhancing the understanding of NNH chemistry in thermal De-NO_*X*_ processes. Complementing this, Hayakawa et al.^54^ conducted a comprehensive experimental investigation into the NO_*X*_ characteristics of ammonia/air flames under varying conditions of equivalence ratio and pressure. The study employed numerical simulations to explore NO formation and reduction mechanisms, identifying the mole fraction of NO as being sensitive to changes in the equivalence ratio and pressure, indicating a decrease in the NO mole fraction with increasing equivalence ratio and pressure. The findings underscore the importance of the third-body reaction of OH + H + M = H_2_O + M in NO reduction at high pressures and highlight the roles of NH_2_, NH, and N in the postflame region of rich mixtures.

**Figure 4:**
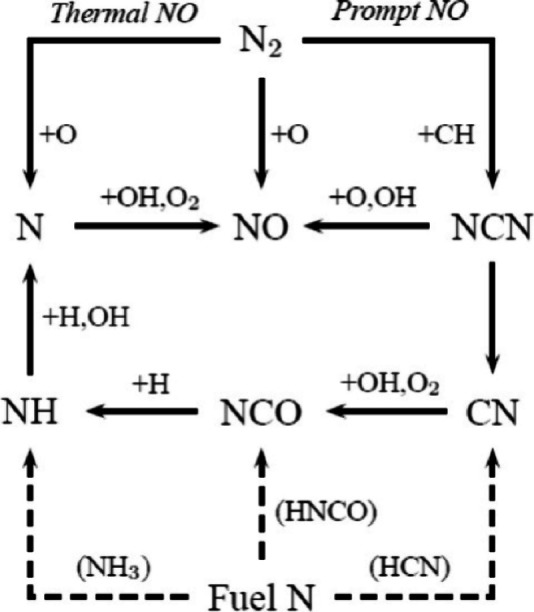
Simplified reaction path diagram illustrating the major steps in the formation of thermal NO, prompt NO, and fuel NO. Reprinted with permission from ref (55). Copyright 2018 Elsevier.

**Figure 5:**
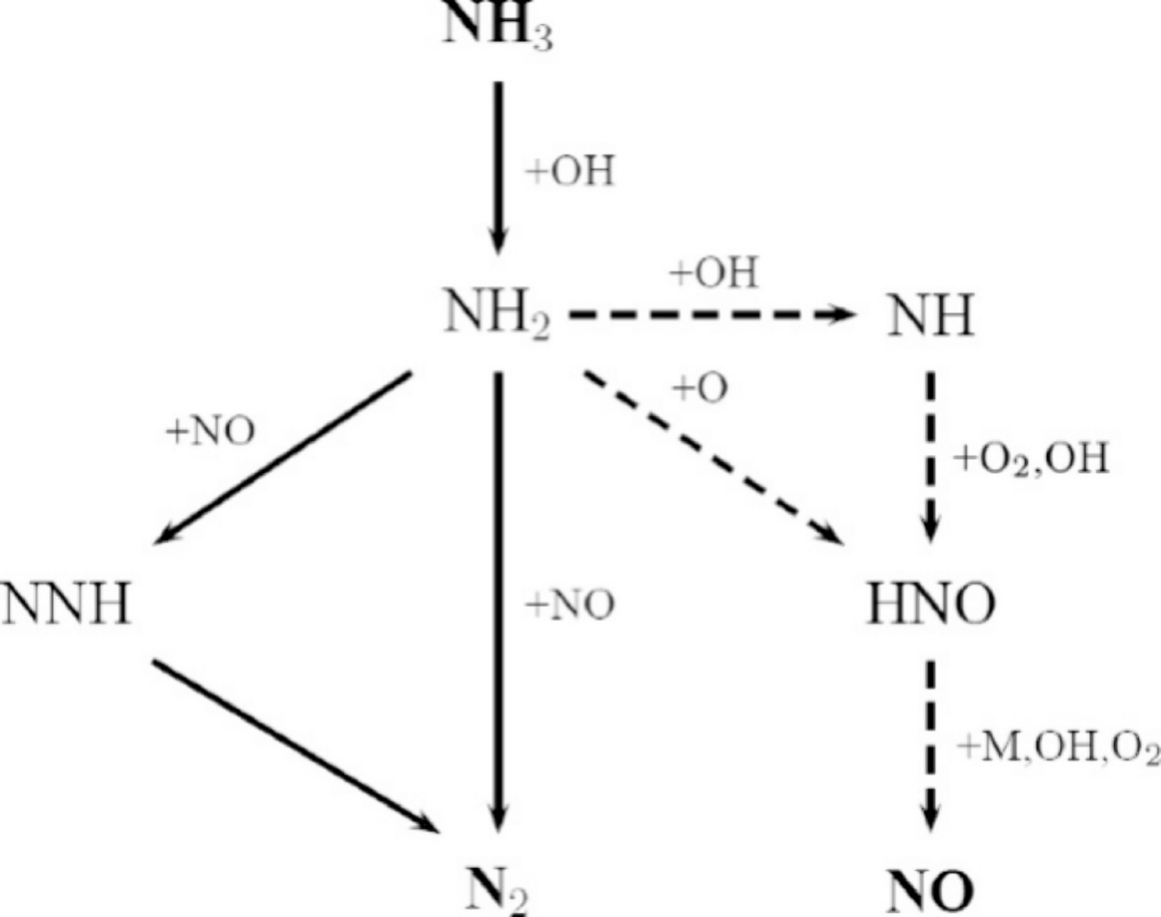
Reaction path diagram for the thermal De-NO_*X*_ process. Dashed lines denote pathways only important at high temperatures. Reprinted with permission from ref (55). Copyright 2018 Elsevier.

Glarborg et al.^55^ offered a detailed review of the various NO_*X*_ formation mechanisms, including thermal NO, prompt-NO, fuel-NO, and NO formation via N_2_O or NNH; a simplified reaction path diagram is shown in Figure 4. The review evaluates these mechanisms against experimental data and discusses the accuracy of modeling predictions. Furthermore, it delves into in situ reduction methods such as humidification, selective noncatalytic reduction, and reburning techniques, which are critical for controlling NO_*X*_ emissions in industrial applications. For example, Figure 5 shows a pathway diagram for the thermal De-NO_*X*_ process in the presence of water vapor. Ammonia is converted to NH_2_ by reaction with the O/H radical pool, primarily OH. The subsequent reaction between NH_2_ and NO is the key step in the process via two product channels: NH_2_ + NO = NNH + OH and NH_2_ + NO = N_2_ + H_2_O.

Pugh et al.^56^ compared swirl-stabilized premixed and diffusion NH_3_–air flames, revealing that the diffusion flame configuration produced significantly lower NO concentrations at lean conditions, while the premixed flame is more favorable at higher equivalence ratios. The study also explored the use of secondary air and reactant humidification as methods to control NO_*X*_ emissions, highlighting the need for careful control to avoid increased NO_*X*_ production. Additionally, Okafor et al.^57^ highlighted the significant influence of wall heat loss on the emission characteristics of premixed ammonia–air swirling flames. Their research shows that wall heat loss can lead to contradictory emission trends, with substantial effects on the flame structure and NO_*X*_ emissions. They advocate for strategies to reduce wall heat loss or extend the primary combustion zone to effectively control NH_3_ and N_2_O emissions effectively. In a related study, Okafor et al.^58^ examined the flame stability and emission control in two-stage micro gas turbine combustors using liquid ammonia spray combustion. Their findings underscore the importance of enhancing the flame in the primary combustion zone and reducing the combustor wall heat loss to control NO_*X*_ emissions.

Enhancing ammonia combustion through oxygen enrichment is a strategic approach to achieving more efficient and cleaner energy conversion, particularly relevant for applications seeking to reduce carbon footprints. Liu et al.^59^ proposed a strategy of using oxygen-enriched conditions to promote self-promoted fuel pyrolysis, which can lead to cleaner and more efficient ammonia combustion. Their findings suggest that oxygen enrichment significantly alters flame characteristics, with flames becoming more compact and stable as the oxygen content increases. This enrichment substantially expands the lean blowout (LBO) and rich blowout (RBO) limits of ammonia swirl flames, indicating a broader stable combustion window. Emissions of NO_*X*_ and NH_3_ were measured, revealing that the low NO_*X*_/NH_3_ emission window also expands with increased oxygen levels, a critical factor for simultaneous pollutant control during combustion enhancement. The self-promoted ammonia pyrolysis under oxygen enrichment emerges as a promising approach for achieving clean and efficient ammonia combustion, effectively broadening both stable combustion and low NO_*X*_/NH_3_ emission windows, which are essential for practical applications in gas turbines, furnaces, and boilers.

Building on this, Kim et al.^60^ conducted a meticulous examination of the influence of oxygen-enriched conditions on the combustion behavior and emission profiles of NH_3_/air premixed flames. They observed that, under elevated oxygen levels, the flames exhibited not only increased propagation speeds but also reduced thicknesses. At a volume fraction of 35–40% O_2_ in the nonfuel mixtures, the flames achieved a burning intensity comparable to that of conventional hydrocarbon/air flames, highlighting NH_3_’s potential as a carbon-free fuel alternative. The research also demonstrated that these O_2_-enriched NH_3_/air flames maintained stability against cellular instabilities at normal temperature and pressure with preferential diffusion playing a negligible role in any destabilizing effects. Despite these promising combustion characteristics, the study highlighted a critical concern: a significant increase in the level of local NO_*X*_ emissions. This finding underscores the necessity for the development of effective strategies to mitigate NO_*X*_ emissions in practical combustion systems employing O_2_-enriched NH_3_/air mixtures, ensuring that the environmental benefits of using ammonia as a fuel are fully realized.

The collective findings from these studies paint a complex picture of the interplay among combustion dynamics, chemical kinetics, and NO_*X*_ emissions in pure ammonia swirl flames. The insights gained are invaluable for developing strategies aimed at minimizing NO_*X*_ emissions while maintaining efficient combustion. As our understanding of these processes continues to evolve, further research is essential to refine these strategies and explore additional methods for controlling NO_*X*_ emissions in practical applications.

#### 2.3.2 Ammonia/Hydrocarbon Fuels

The swirl flames of ammonia and hydrocarbon fuels present a rich tapestry of chemical kinetics and thermodynamic interactions that significantly influence NO_*X*_ emissions. This section endeavors to weave together the findings from various studies, elucidating the multifaceted nature of this challenge and the strategies devised to address it.

The pioneering works of Tian et al.^61^ and Mendiara and Glarborg^62^ shed light on the combustion dynamics of ammonia–hydrocarbon mixtures and the mechanisms that govern the NO_*X*_ emissions. Tian et al.^61^ focused on the combustion of ammonia with methane under low-pressure conditions and illuminated the role of NH_2_ and NH radicals in the selectivity of NO or N_2_ formation, identifying key reactions that govern NO_*X*_ generation. Notably, the reactions NH_2_ + O = HNO + H and NH + NO = N_2_ + O are identified as crucial in the generation of NO_*X*_. They discovered that, as the NH_3_ to CH_4_ mole ratio increases, the reaction zone expands and the levels of H_2_O, NO, and N_2_ rise, while H_2_, CO, CO_2_, and NO_2_ decrease. This suggests that higher ammonia concentrations may lead to greater NO_*X*_ emissions. Additionally, they found that the temperature profiles of the flames decrease with an increased NH_3_/CH_4_ mole ratio due to the reduction of CH_4_, which can affect the formation and reduction pathways of NO_*X*_. The reactions H + O_2_ = O + OH and NH_2_ + O = HNO + H emerge as significant contributors to NO formation, whereas NH_2_ + NO = N_2_ + H_2_O and NH + NO = N_2_O + H are instrumental in NO consumption. Mendiara and Glarborg,^62^ on the other hand, investigated the impact of high CO_2_ concentrations on ammonia oxidation during the oxy-fuel combustion of methane. Their findings indicate that high CO_2_ levels enhance NO formation under reducing conditions but inhibit it under stoichiometric and lean conditions. The presence of CO_2_ as a bulk gas was found to facilitate NO formation due to an increased OH/H ratio and higher CO levels that enhance HNCO formation, while reactions leading to HNO and NH are inhibited under high CO_2_ levels due to reduced concentrations of O and H radicals, leading to an increased probability of forming N_2_ instead of NO.

Building upon these foundational studies, subsequent research has delved deeper into the factors influencing NO_*X*_ emissions. Valera-Medina et al.^63^ explored the emission characteristics of premixed ammonia–methane swirling flames, noting a reduction in NO_*X*_ and CO_2_ emissions with increasing equivalence ratio, while CO, THC, and unburned NH_3_ emissions increased. Xiao et al.^64^ expanded on this by examining methane–ammonia flames across a wide range of mixing ratios, finding that the addition of ammonia to methane-rich mixtures enhances NO_*X*_ emissions, while high ammonia content in fuel mixtures has a De-NO_*X*_ing effect. They also noted that fuel-bound NO_*X*_ formation is more sensitive to ammonia content than thermal NO_*X*_, particularly in regions with a low ammonia content. Li et al.^65^ and Okafor et al.^66^ further highlighted the critical factors influencing NO_*X*_ emissions in NH_3_/CH_4_ combustion. For example, Li et al.^65^ highlighted the HNO pathway as the predominant driver of NO_*X*_ formation in NH_3_/CH_4_ combustion, with a sharp increase in NO_*X*_ emissions observed with NH_3_ addition up to a 20% dilution. They also emphasized the effectiveness of air staging in reducing NO_*X*_ emissions. Okafor et al.^66^ underscored the role of OH radicals in fuel NO_*X*_ production and advocated for a rich-lean combustion strategy to achieve low emissions. Moreover, Zhang et al.^67^ reported nonmonotonic NO and NO_2_ emissions trends for different methane–ammonia blends, with the highest NO emissions observed for a 50/50 vol % CH_4_/NH_3_ blend at stoichiometry. They suggested operating the combustor under rich conditions and avoiding an NH_3_ mole fraction of 0.5 to control the NO_*X*_ emissions.

**Figure 6:**
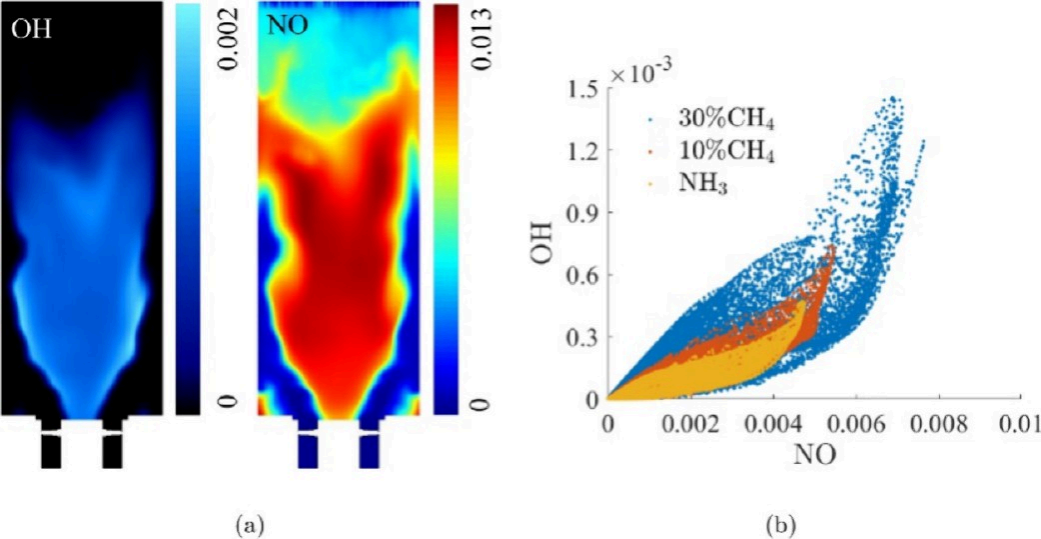
(a) Instantaneous distributions of the NO and OH mass fractions of the ammonia flame in the combustor. (b) Relationship of the local NO and OH mass fractions. The flame is operated at ϕ = 0.7 and *U* = 5 m/s. Reproduced with permission from ref (69). Copyright 2021 Elsevier.

Subsequently, An et al.^68^ and Zhang et al.^69^ investigated the effects of methane and hydrogen additives on NO_*X*_ emissions. To be specific, An et al.^68^ found that cofiring methane with ammonia in premixed swirling flames increases NO_*X*_ emissions, with a peak at a 40% NH_3_ mole fraction. Zhang et al.^69^ examined the effects of methane and hydrogen additives on NO_*X*_ emissions in ammonia/air flames within a swirl combustor, finding that small amounts of these additives can stabilize the flame without significantly increasing NO_*X*_ emissions. A positive correlation between OH and NO concentrations is displayed in Figure 6, indicating a temperature-dependent relationship.

Early studies^70,71^ identified reactions such as CH + N_2_ = HCN + N and CH + N_2_ = NCN + N as critical initiation steps toward prompt NO formation. NCN eventually reacts with H radicals to convert into HCN, which then reacts with the O radicals to produce NCO. NCO converts into NH radicals that produce atomic nitrogen. Glarborg et al.^55^ reported a rapid increase in HCN concentration at rich conditions (ϕ > 1.2), highlighting the role of amine radicals and NO recycling back to HCN.

The influence of the pressure and temperature on NO_*X*_ emissions has been a subject of interest for the NH_3_/CH_4_ swirl flame. The study by Xiao et al.^72^ has shown that increased pressure leads to a reduction in NO and CO emissions, while increased initial temperature results in an augmentation of emissions. As the methane mole fraction in the fuel mixture increases, the NO_*X*_ concentration in the flame also increases. This trend is consistent across different equivalence ratios, indicating that methane acts as a promoter for NO_*X*_ formation. Sensitivity analyses were conducted to identify key reactions impacting NO_*X*_ conversion. The results show that the reactions H + O_2_ = O + OH (promoting) and NH_2_ + NO = N_2_ + H_2_O and NH + NO = N_2_O + H (inhibiting) play significant roles in NO_*X*_ chemistry across different fuel compositions and equivalence ratios. The presence of methane in ammonia fuels leads to enhanced combustion intensities, as indicated by increased temperatures, heat release rates, and concentrations of important intermediate radicals such as OH, H, and O. The pressure is identified as a more significant factor affecting the NO_*X*_ kinetics under practical engine operational conditions. Somarathne et al.^73^ investigated the impact of OH concentration and temperature on NO_*X*_ emissions in turbulent nonpremixed methane/ammonia/air swirl flames at high pressure. Their study indicated that adjusting the equivalence ratios to far-rich conditions could significantly reduce the level of NO_*X*_ emissions. They also noted that local NO and OH concentrations and temperature distributions were similar to those in NH_3_/air flames, with the highest NO_*X*_ emissions occurring at an energy fraction of NH_3_ between 20 and 30%.

Recently, Mashruk et al.^74^ investigated methane/ammonia/hydrogen ternary blends in a swirl burner. They observed minimum N_2_O and NO_2_ emissions under rich conditions, with significant NO emissions at high methane mixtures due to the availability of OH radicals. The study also noted that NH_3_ slip increased initially with decreasing methane content but then dropped as the hydrogen content increased, indicating the dominance of hydrogen chemistry.

The use of DME as a combustion promoter in ammonia flames is advantageous due to its high reactivity and ability to enhance flame stability and reduce emissions. Meng et al.^46^ found that fuel NO_*X*_ dominates the total NO_*X*_ production in NH_3_/DME combustion. Adding up to 50% DME to ammonia promotes the NO formation reaction, leading to higher NO production. The study found that a higher ammonia content in the blend (as in D25, 75% NH_3_/25% DME) generates more NH and NH_2_ radicals, promoting the NO reduction reaction. Lower DME content also results in fewer H/O/OH active radicals, inhibiting NO generation. Yu et al.^75^ applied the fuel staging method to mitigate the issue of NO_*X*_ emissions of the NH_3_/DME/air flame. The NO removal efficiency initially increased with temperature and then decreased, with optimal reaction temperatures at 950 °C for an equivalence ratio of the primary stage (ϕ_pri_) at 0.9 and 900 °C for ϕ_pri_ at 0.75. The NO removal efficiency improved with longer residence times and increased the number of secondary NH_3_ injections. NH_3_ slip was significant at lower temperatures but decreased dramatically above 950 °C due to NH_3_ oxidation. The fuel staging method, with optimization of parameters like temperature, residence time, and NH_3_ injection, can significantly reduce NO_*X*_ emissions from NH_3_/DME cocombustion, with a maximum NO removal efficiency of 55.2% achieved in this study.

Lian et al.^76^ investigated the impact of CO_2_ exhaust gas recirculation (EGR) on the flame characteristics and NO_*X*_ emissions of premixed NH_3_/DME swirl flames. CO_2_ EGR significantly affects flame morphology, making the flame weaker and increasing its height, which indicates reduced flame stability. The lean blowout (LBO) limit increases with an increasing CO_2_ EGR rate, suggesting that higher CO_2_ concentrations make the flame less stable. CO_2_ EGR reduces the maximum mole fraction of the OH radical, affecting the flame’s chemiluminescence intensity and distribution. NO_*X*_ emissions decrease with an increasing CO_2_ EGR rate due to the thermal effect of CO_2_, which reduces the flame temperature and thus the formation of thermal NO_*X*_. Yu et al.^47^ investigated the effects of partial precracking of NH_3_ and the use of DME as a combustion promoter on flame macrostructures, LBO characteristics, and exhaust emissions, including NO_*X*_, CO, and unburned NH_3_. As the precracking ratio of NH_3_ increases, the flame height shortens, OH fluorescence intensifies, and core jet velocities amplify, leading to a significant reduction in the LBO limit, indicating enhanced combustion. NO and NO_2_ emissions increase with a larger precracking ratio, while CO and unburned NH_3_ emissions increase sharply under fuel-rich conditions due to insufficient oxygen. The study reveals a trade-off between NO and NH_3_ emissions, with relatively low NO/NH_3_ emissions observed under slightly rich conditions (ϕ = 1.0–1.1). The partial precracking strategy effectively enhances NH_3_ combustion, but it also increases NO_*X*_ emissions. The study suggests that burning the partially precracked NH_3_ directly rather than separating N_2_ from the mixtures is more feasible due to deteriorating effects on NO_*X*_ emissions.

Moreover, unburned ammonia emissions are a critical aspect of the emissions profile for combustion systems. Ammonia at the exhaust can lead to the formation of particulate matter via ammonium sulfides and nitrates, thus impacting the environmental quality. It is essential to design combustion systems that mitigate ammonia slip to avoid nuisances and reduce particulate matter.

This section underscores the multifaceted nature of NO_*X*_ emissions in the swirl flames of ammonia and hydrocarbon fuels. It highlights the critical interplay between fuel composition, chemical kinetics, combustion dynamics, and the environment. The findings provide a foundation for developing advanced combustion strategies and emission control technologies, ensuring sustainable and efficient energy systems.

#### 2.3.3 Ammonia/Hydrogen Fuels

The quest for decarbonization in the power sector has led to the exploration of ammonia–hydrogen blends as cleaner alternatives to fossil fuels. The combustion of these blends, particularly in swirl flame configurations, presents unique challenges and opportunities for NO_*X*_ emission control. Initial studies by Joo et al.^77^ and Li et al.^78^ established the potential of ammonia substitution to enhance the safety of hydrogen use. They observed a decrease in NO_*X*_ production with an increased ammonia slip as flames transitioned from stoichiometric to rich conditions. This reduction was attributed to lower flame temperatures, which favor NO_*X*_ reduction mechanisms. Valera-Medina et al.^79^ extended the research by examining lean premixed combustion of a 50/50 vol % NH_3_/H_2_ blend in swirling flames. Their findings indicated a significant reduction in NO_*X*_ emissions with decreasing equivalence ratios, achieving as low as ∼100 ppm wet NO at ϕ = 0.40.

A pivotal sensitivity analysis by Tian et al.^61^ and Xiao et al.^80^ identified the reaction NH_2_ + O = HNO + H as a key promoter of NO formation, highlighting the influence of increasing ammonia concentration in the fuel blend on NO exhaust concentrations. Further studies by Xiao et al.^81^ revealed that NO_*X*_ emissions decrease with increasing pressure, suggesting that the use of ammonia/hydrogen blends in gas turbines could result in reduced NO_*X*_ emissions under practical operating conditions. This finding underscores the potential of pressure as a key parameter in optimizing combustion processes for lower emissions.

**Figure 7:**
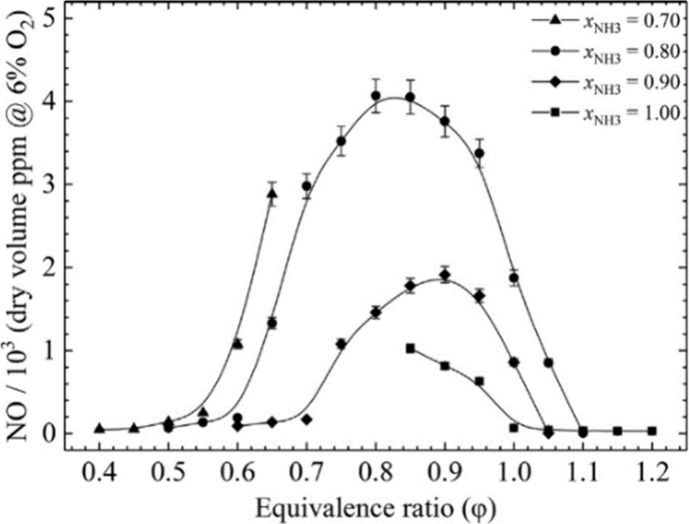
Measured exhaust NO mole fraction in ammonia–hydrogen–air flames in parts per million (ppm) as a function of equivalence ratio for *X*_NH_3__ = 0.70 (triangles), 0.80 (circles), 0.90 (diamonds), and 1.00 (squares) for *S*_g_ = 1.00 and *Re* = 5000. Results are normalized for a 6% O_2_ mole fraction. Reproduced with permission from ref (82). Copyright 2020 Elsevier.

**Figure 8:**
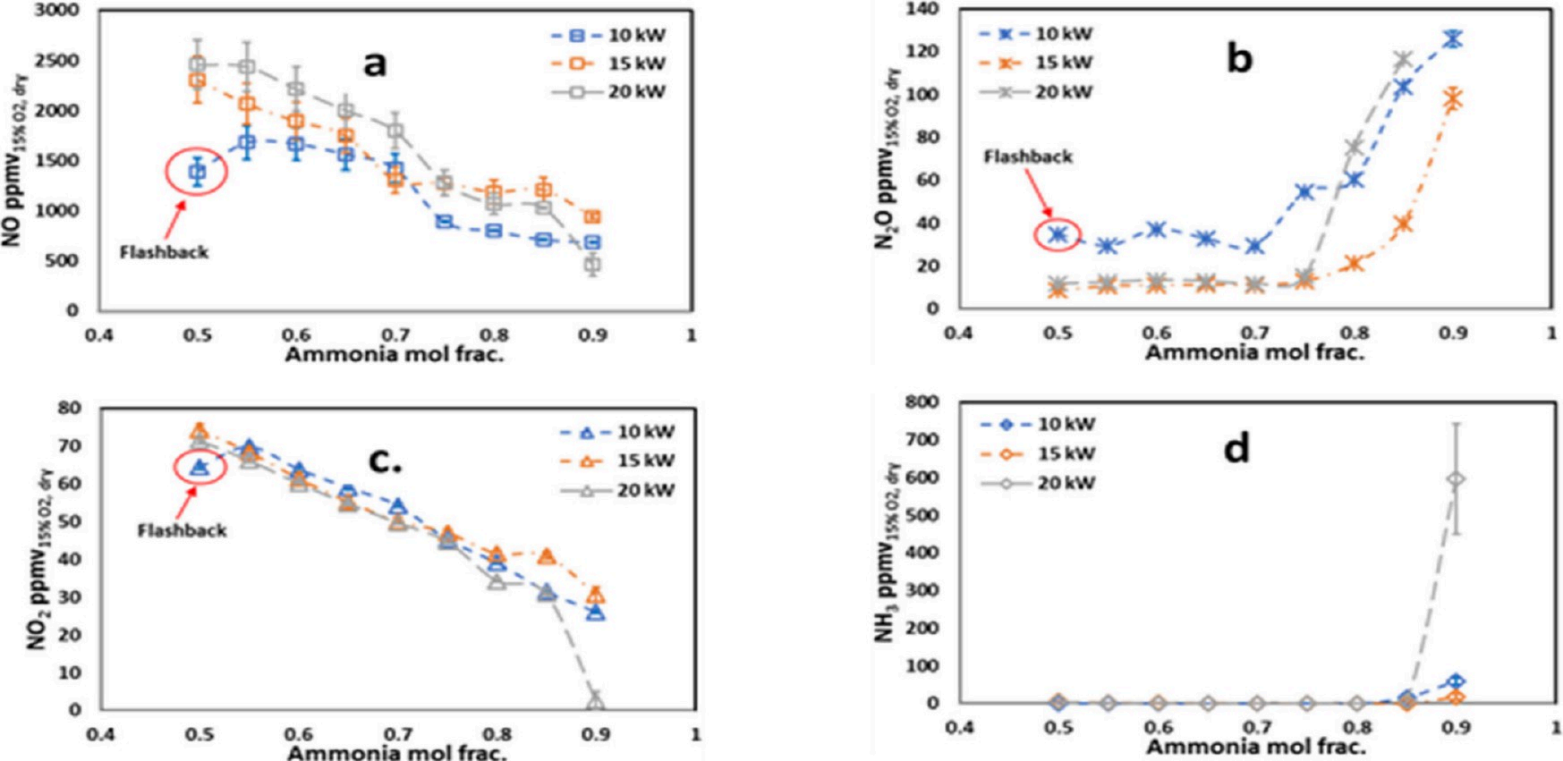
Sampled NO (a), N_2_O (b), NO_2_ (c), and NH_3_ (d) emissions at different thermal powers and ϕ = 0.65. From ref (83). CC BY 4.0.

Further investigations were made by Khateeb et al.^82^ and Zhu et al.^18^ into various lean ammonia/hydrogen swirling flames at *Re* = 5000 and under atmospheric conditions. These studies showed significant NO reduction under lean conditions for low turbulent ammonia–hydrogen flames, as shown in Figure 7. However, they did not address NO_2_, N_2_O, and NH_3_ emissions, leading to the comprehensive study by Mashruk et al.^83^ where the authors have investigated different ammonia/hydrogen blends at a fixed lean equivalence ratio (ϕ = 0.65) with different thermal powers and *Re* under atmospheric conditions (Figure 8). They reported a decrease in NO and NO_2_ emissions but an increase in N_2_O production with a higher ammonia content in the fuel mixtures. This trend was more pronounced at elevated thermal powers or Reynolds numbers, highlighting the role of radical formation rates, particularly NH, OH, and NH_2_. The reaction NH + NO = N_2_O + H was pinpointed as the primary N_2_O source in the flame, with postflame N_2_O consumption occurring primarily through N_2_O + H = N_2_ + OH and N_2_O (+M) = N_2_ + O (+M).

**Figure 9:**
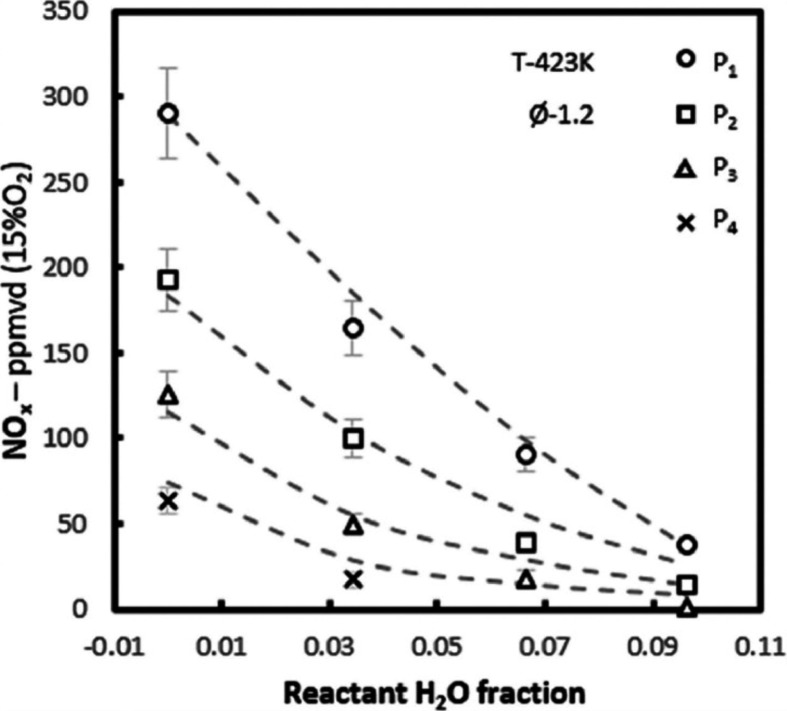
Experimental (symbols) and modeled (lines) NO_*X*_ concentrations against reactant water fraction with increased pressure (Ø = 1.2). From ref (84). CC BY 4.0.

The influence of humidification on NO_*X*_ production was initially explored by Pugh et al.^84^ in NH_3_/H_2_ flames. They found that the addition of steam reduced NO_*X*_ concentrations (see Figure 9) by limiting thermal pathways and enhancing NO consumption in the postflame zone. The application of staged combustion, with secondary airflow, improved fuel burnout and further reduced NO_*X*_ emissions. An increase in combustor pressure up to 0.185 MPa resulted in a significant reduction in NO_*X*_ concentrations, primarily due to enhanced NH_2_ formation, which subsequently consumed NO in the postflame zone. The research demonstrates that NO_*X*_ emissions from premixed swirling NH_3_/H_2_ flames can be significantly reduced through a combination of elevated pressure, reactant humidification, and staged combustion techniques. However, a careful balance is needed to avoid increasing unburned NH_3_, which could eventually lead to higher NO_*X*_ formation in the secondary reaction zone.

**Figure 10:**
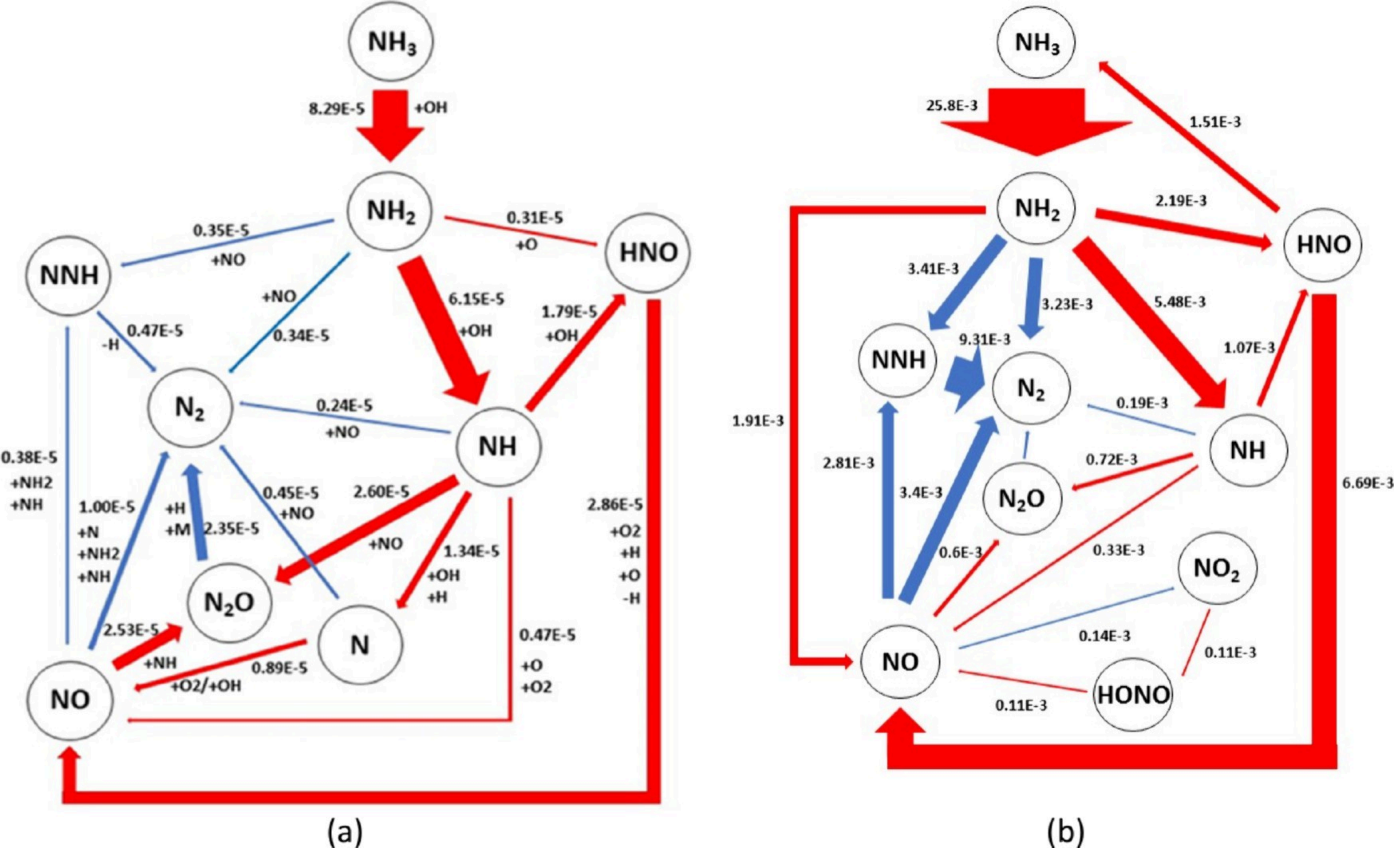
(a) Fuel NO_*X*_ formation and reburn pathways under lean conditions (ϕ = 0.65). Reproduced from ref (83). Copyright 2022 Elsevier. (b) Fuel NO_*X*_ formation and reburn pathways under rich conditions (ϕ = 1.20). Reproduced with permission from ref (85). Copyright 2021 Elsevier.

Moreover, the potential of humidified ammonia/hydrogen systems using rich-quench-lean (RQL) technology was investigated by Mashruk et al.,^85^ demonstrating its efficacy in emission reduction at high power outputs. Sensitivity analyses identified HONO, HNO, and NO_2_ as critical species for NO formation, with NH_2_ playing a vital role in NO consumption. The interplay of chemical reactions in ammonia/hydrogen combustion was found to be complex, but the RQL system’s combination with humidified atmospheres effectively lowered emissions through species recombination and reduced combustion temperatures. As shown in Figure 10, these two studies illustrated show the NO_*X*_ formation/reburn routes in a 70/30 vol % NH_3_/H_2_ blend studied by Mashruk et al. at lean (ϕ = 0.65)^83^ and rich conditions (ϕ = 1.20),^85^ respectively, in swirling turbulent flames. Ammonia reacts with OH radicals to produce NH_2_ radicals in both rich and lean conditions, which converts to HNO directly by reacting with O radicals and via NH radicals by reacting with OH. Nitroxyl (HNO) is the main source of fuel NO production in ammonia flame through reactions with molecular oxygen and H and O radicals, as well as through disassociation processes.^31^

Subsequent studies^13,86^ recommended an optimal equivalence ratio of 1.20 for two-stage burner configurations, balancing emissions performance and combustion efficiency. The reactions involving NH radicals were also highlighted as significant contributors to NO and N_2_O production, with the latter being predominantly converted to N_2_ through reactions with H radicals and the third-body reaction N_2_O (+M) = N_2_ + O (+M). These reactions, along with the conversion of N_2_O to N_2_, are critical in managing NO_*X*_ emissions in ammonia/hydrogen swirl flames. Other prominent sources of NO reduction are through the chain branching reaction NH_2_ + NO = NNH + OH and the terminating reaction NH_2_ + NO = N_2_ + H_2_O.^87,88^

Moreover, Shi et al.^89^ studied the effects of partial precracking of ammonia on flame macrostructures, lean blowout (LBO) characteristics, and exhaust emissions in a gas turbine model combustor. The results demonstrated that increasing the precracking ratio (γ) leads to a more compact and stable flame, as evidenced by the shorter flame height, intensified OH fluorescence, and amplified core jet velocities. Consequently, the LBO limit is significantly reduced, indicating enhanced NH_3_ combustion. However, the trade-off between NO and NH_3_ emissions becomes more pronounced with higher precracking ratios, as NO and NO_2_ emissions increase substantially while NH_3_ emissions decrease. The study also found that separating N_2_ from the partially precracked NH_3_ mixtures can lead to increased NO_*X*_ emissions, suggesting that direct burning of the partially precracked NH_3_ is a more favorable approach. Overall, the partial precracking strategy presents a promising avenue for improving NH_3_ combustion characteristics in gas turbine model combustors, but careful consideration must be given to the trade-off between NO_*X*_ and NH_3_ emissions.

In summary, this section offers a comprehensive understanding of the chemical kinetics governing the NO_*X*_ emissions in ammonia/hydrogen swirl flames. By integrating these findings into a cohesive narrative, we can better grasp the interplay of factors influencing NO_*X*_ emissions and develop targeted strategies to mitigate these emissions in practical combustion systems.

#### 2.3.4 Ammonia–Coal Cofiring

Ammonia–coal cofiring is also a promising technology that large power generation companies are evaluating mainly in Asia. Replacement of coal appears to have many benefits related not only to CO_2_ abatement but also to mitigation of nitrogen oxide emissions and higher radiation outputs. Cui et al.^90^ investigated NO_*X*_ (i.e., NO, N_2_O, NO_2_) emissions from ammonia–coal cofiring using different pulverized coal concentrations. It was found that the reducing effect of unburned ammonia led to the rapid decrease of N_2_O in the last stages of combustion, while the combustion of char caused the reduction of NO_2_. The results also demonstrated that when coal was decreased, the oxygen concentration increased with the formation of NO and NO_2_.^91^ Further studies also elucidated the importance of water content which after decomposition produced large pools of OH radicals, capable of impacting the reconversion of NO_*X*_ species.^92^

Fan et al.^93^ used a fixed-bed reactor for their studies on the pore surface impact on NO_*X*_ emissions using ammonia–coal blends. Chars with the largest porosity showed a maximum NO reduction efficiency of >90%, while smaller porosities denoted lower reduction efficiencies of ∼80%, hence with the finding that enriched micropores with large pore volume and surface area could increase the adsorption capacity of coal. Therefore, more ammonia could be adsorbed on the surface of the coal, hence promoting overall NO_*X*_ reduction reactions. Further studies by the group also showed that using additives can have an impact on NO mitigation, with Fe_2_O_3_, Fe_3_O_4_, and Cu_2_O in the fly ash having an improved increase in NO reduction efficiency by nearly 30%. However, Chen et al.^94^ showed that Fe can have adverse impacts on the reduction of NO due to the potential absorption of NH_*X*_ free radicals that promote NO consumption.

**Figure 11:**
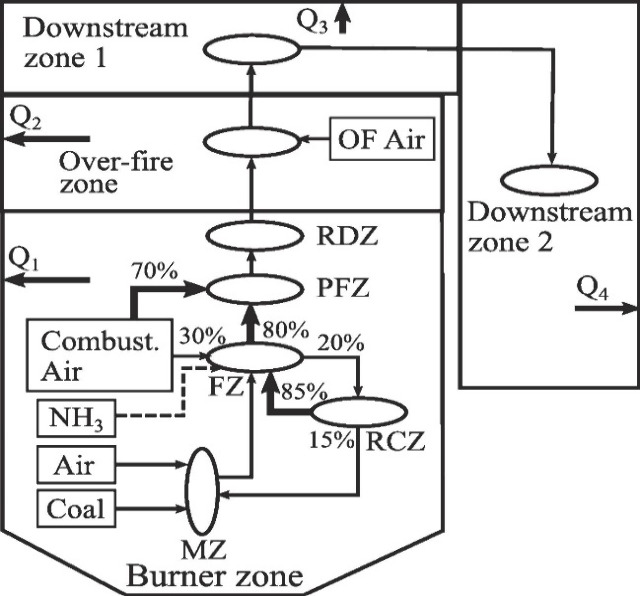
Reactor network representing a pulverized coal fired boiler with NH_3_ cofiring. MZ, mixing zone; FZ, flame zone; RCZ, recirculation zone; PFZ, postflame zone; RDZ, reduction zone; OF Air: overfire air. Q1–Q4: local heat absorption in each zone. Reprinted with permission from ref (96). Copyright 2020 Elsevier.

**Figure 12:**
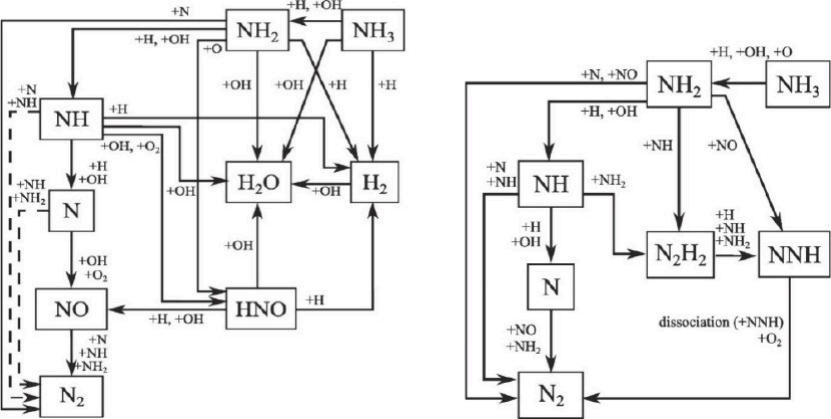
Comparison between ammonia–coal reactions at (left) 20 and 80% (dashed line) and (right) 40 and 60% ammonia contents. Reprinted with permission from ref (96). Copyright 2020 Elsevier.

Attention has also been paid to the ammonia–coal ratios. A reactor network, representing an IHI burner (Figure 11) and three different modes of ammonia injection, was used for that purpose (Figure 12). Results found that low CO_2_ and NO_*X*_ could be produced by the reduction of coal and various processes such as ammonia De-NO_*X*_ing, lower temperatures, and char–NO reducing effects, respectively. Additional work^95^ showed that the increase of water content from ammonia combustion could also reduce CO by the water gas shift reaction, H_2_O + CO → H_2_ + CO_2_.

The work also addressed the difference in reaction paths between the 20 and 80% cases with the 40 and 60% scenarios, as shown in Figure 12. NO emissions are considerably reduced at 20% due to postcombustion reactions, while at 80% there are more effective reactions that do not involve NO production and that lead to N_2_ via NH_*i*_ recombination. Although 40 and 60% NO at the flame are kept low via reactions that produce N_2_H_2_ and NNH, the higher temperatures in the postcombustion zone elevate thermal NO_*X*_.

Fundamental analyses of the temperature dependency of NO have also been conducted when coal is cofired with ammonia. Jiang et al.^97^ used a high-temperature fixed-bed reactor and conducted ammonia/coal cofiring experiments. Results showed that between 0 and 10% blending ratios, as the temperature increases, NO_*X*_ reaches a peak in a shorter time. An interesting pattern is that the peak fluctuates, from low NO_*X*_ emissions at ∼1000 °C, peaking at 1200 °C, to then decay and reach minimum values at 1500 °C. This behavior is believed to be caused by the increase of NH_*i*_ free radicals, hence contributing to the reduction of NO. Similarly, the reduction of oxygen would have an impact on NO_*X*_ emissions, as previously described in other types of flames. Niu et al.^98^ showed that the reduction in oxygen content has a remarkable impact on the reduction of NO. Analyses showed that a decrease from 4.24 to 2.35% causes a drop in up to 36% nitrogen emissions. Furthermore, Fan et al.^93^ found that NH_3_ has a De-NO_*X*_ing effect under oxygen-depleted atmospheres. The studies showed that, at 0% oxygen content and 1300 °C, the highest reduction efficiency was close to 50%.

The impact of different fuels on NO_*X*_ emissions in ammonia swirl flames varies significantly based on fuel composition and combustion conditions. Pure ammonia flames produce high NO_*X*_ emissions due to thermal NO_*X*_ formation at high temperatures, with radicals such as NH and NH_2_ playing a crucial role. While hydrogen addition can generally reduce NO_*X*_ emissions, especially under lean conditions, hydrocarbons like methane and DME can have more complex effects. Ammonia/methane blends initially increase NO_*X*_ emissions with added ammonia, but higher concentrations may reduce the level of NO_*X*_ due to enhanced reduction pathways. CO_2_ as a diluent generally reduces NO_*X*_ emissions under lean conditions. Ammonia/DME blends show increased NO_*X*_ emissions with added DME due to its high reactivity, though higher ammonia content can reduce NO_*X*_ through enhanced reduction reactions. Fuel staging and optimized combustion parameters can significantly reduce the level of NO_*X*_ in NH_3_/DME flames. Ammonia/hydrogen blends offer lower NO_*X*_ emissions, especially at lean conditions. However, the formation of other nitrogenous species such as N_2_O must be managed. Each fuel blend presents unique advantages and challenges, necessitating optimized combustion parameters to achieve low NO_*X*_ emissions.

### 2.4 Plasma-Assisted Combustion

In the literature, various strategies have been explored to address the high NO_*X*_ and N_2_O emissions of ammonia combustion. A notable method involves blending ammonia (NH_3_) with hydrogen (H_2_), which improves the burning velocity owing to hydrogen’s higher mass diffusivity.^5^ While this approach mitigates some of the issues inherent in traditional NH_3_ combustion, hydrogen production itself poses challenges. Additionally, increasing hydrogen’s volume fraction from 0 to 30% in fuel-lean mixtures can significantly escalate NO_*X*_ emissions. It has also been observed that combusting NH_3_/H_2_ blends under extremely lean conditions can induce thermoacoustic instabilities, flame instabilities, and elevated emissions of NO and N_2_O.^8^ Over the past decade, nonequilibrium (or nonthermal/cold) plasma technology has shown substantial promise in boosting ignition, expanding flammability limits, accelerating low-temperature oxidation, and cutting emissions in combustion.^99,100^ This technology leverages the disparity between the translational and internal degrees of gas molecules and electrons to facilitate robust momentum and energy transfer at lower temperatures, effectively exciting and dissociating target molecules with minimal heat loss or energy inefficiency. Furthermore, nonequilibrium plasma enhances combustion by generating chemically reactive species at low temperatures, such as high-energy electrons, excited species, and ions. The degree to which nonequilibrium plasma enhances combustion is heavily influenced by specific plasma properties, such as electron temperature and electron number density. These critical plasma characteristics are determined by the reduced electric field (*E*/*N*), which represents the ratio of the electric field strength to the molecular number density. The subject, novel and with limited published literature, opens the possibility of using advanced systems to control and mitigate NO_*X*_ emissions in ammonia flame systems.

**Figure 13:**
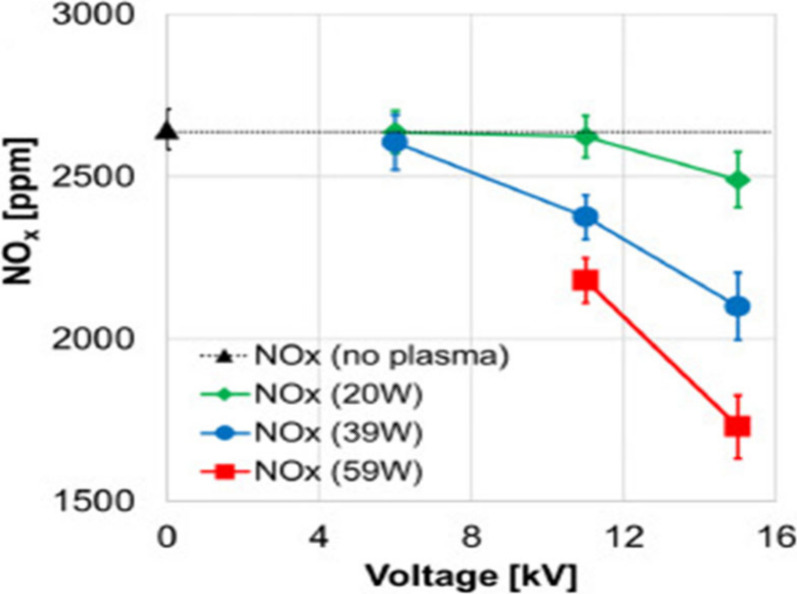
NO_*X*_ variation with discharge power and discharge voltage. Reproduced with permission from ref (103). Copyright 2021 Elsevier.

There are two recent review papers^101,102^ that extensively discuss plasma-assisted ammonia combustion, though we will not go into detail on these here. All the experimental and numerical studies on plasma-assisted ammonia combustion give clear evidence that plasma-assisted ammonia flames are feasible to improve the combustion process while producing less NO_*X*_ compared to conventional ammonia flames. However, the level of NO_*X*_ mitigation can be affected by a series of factors. Some that have been identified so far are plasma discharge gas, plasma type, equivalence ratio, and plasma conditions (applied voltage and pulse repetition frequency). Choe et al.^103^ addressed the variation of NO_*X*_ concentrations with discharge voltage and discharge power at a constant equivalence ratio, ϕ = 0.94, as shown in Figure 13.

**Figure 14:**
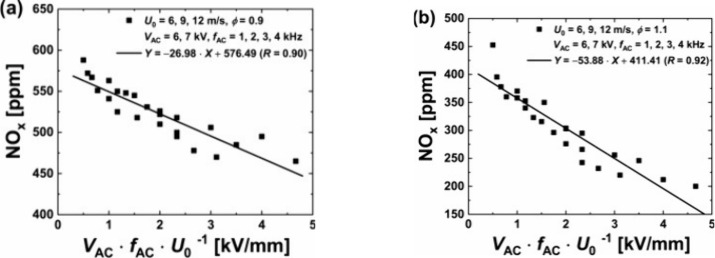
Correlation between NO_*X*_ and *V*_AC_·*f*_AC_·*U*_0_^–1^ for (a) ϕ = 0.9 and (b) ϕ = 1.1. Reproduced with permission from ref (106). Copyright 2022 Elsevier.

NO_*X*_ concentrations were reduced with nanosecond pulsed discharge plasma, and further reductions were achieved with the increase of both discharge voltage and discharge power. The NO_*X*_ suppression was linked to the reaction of NO with abundant NH_2_* generated by plasma through the thermal De-NO_*X*_ process.^104^ Tang et al.^105^ reported the limited effect of plasma on NO_*X*_ (NO and NO_2_) emissions in a range of equivalence ratios from 0.76 to 1.00, due to the added effect of thermal NO emission induced by the gliding arc discharges. However, the effect became more prevalent in lean ammonia flames, where NO_*X*_ emissions dropped to less than 100 ppm at ϕ = 0.57. The authors discussed the reduction of the possible reaction of unburnt NH_3_ with NO_*X*_. Kim et al.^106^ extended the investigation of plasma-flame coupling to rich ammonia flames. It was found that the NO_*X*_ suppression with and without plasma remained negligible at fuel-rich conditions, ϕ = 1.2–1.3, and became remarkable at ϕ ≤ 1. By applying plasmas, NO_*X*_ concentrations dropped from 691 to 487 ppm at ϕ = 0.9. The authors also found a negative correlation between NO_*X*_ concentration and the product of discharge voltage, applied frequency, and the inverse of the flow residence time, implying that NO_*X*_ could be further reduced by increasing discharge power and by reducing the residence time, as shown in Figure 14.

Lin et al.^107^ reported that plasmas could reduce NO emissions below 100 ppm in lean ammonia flames when air served as a discharge gas. In the case of ammonia being used as a discharge gas, plasma-assisted ammonia flames produced NO concentrations below 100 ppm (at a particular flow rate), regardless of the equivalence ratio. The authors speculated that applying discharges to the ammonia stream might produce a rich mixture of H*, NH*, and NH_2_* that play a vital role in NO consumption.

Recently researchers at Princeton University for the first time studied plasma-assisted ammonia oxidation at room temperature with a focus on unveiling nonequilibrium NO_*X*_/N_2_O reaction pathways by combining in situ laser diagnostics using a miniature plasma reactor and kinetic plasma modeling using the 0D hybrid ZDPlasKin–CHEMKIN solver.^108,109^ Their study revealed that nonequilibrium plasma regulates NO_*X*_ production through the generation of O/H/N atoms from electron-impact dissociation and the quenching of excited states. N_2_O is produced via a dual-step process, beginning with the formation of amine radicals by electron-impact reactions, which then combine with NO_*X*_ to form N_2_O. This process leads to efficient and eco-friendly ammonia oxidation, enhancing reactivity and minimizing emissions of NO_*X*_ and N_2_O by optimizing the mixture compositions and plasma parameters. Findings demonstrate that plasma discharge notably boosts ammonia ignition at lower temperatures and decreases ignition delay, especially under fuel-lean conditions, in contrast to fuel-rich autoignition scenarios. They outlined three approaches to optimize plasma operations for effective ammonia oxidation and reduced pollutant emissions based on nonequilibrium N_2_O/NO_*X*_ chemistry. First, using fuel-rich conditions was recommended for plasma-aided low-temperature ammonia oxidation to maximize electron energy directed toward ammonia dissociation. Second, it emphasized the importance of maintaining an optimal *E*/*N* ratio to ensure efficient energy use primarily for ammonia dissociation, avoiding energy losses to nitrogen excitation or dissociation. Lastly, it suggested that modulating the discharge frequency can help control the formation and transformation of intermediate species, optimizing the chemical reaction pathways.

**Figure 15:**
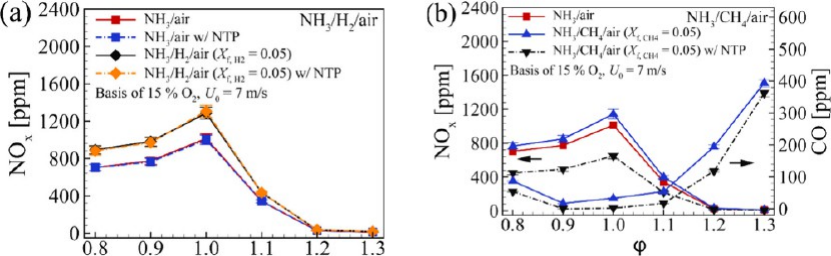
Variations in NO_*X*_ emission as a function of ϕ for NH_3_/air NH_3_/H_2_/air and NH_3_/CH_4_/air premixed flames with and without NTP. Reproduced with permission from ref (110). Copyright 2024 Elsevier.

In the context of the gas turbine setting, Kim et al.^110^ explored the synergetic effects of nonthermal plasma and methane (CH_4_) addition on enhancing ammonia/air premixed flames and NO_*X*_/CO emission characteristics. They experimentally investigated the changes in flame behavior by varying the equivalence ratio, mixture velocity, and methane content in the fuel mix. The NO_*X*_ and CO emissions in NH_3_/air, NH_3_/H_2_/air, and NH_3_/CH_4_/air flames with/without nonthermal plasma (NTP) are shown in Figure 15. It can be seen that the addition of CH_4_ notably intensifies streamer generation compared to H_2_, primarily due to the role of positive ions from CH_4_ in streamer formation. This intensified streamer activity not only improves ammonia combustion when combined with CH_4_ but also significantly broadens the lean blowout limits of NH_3_/CH_4_/air flames compared with those without NTP application. The study also demonstrates that streamer intensity correlates linearly with the equivalence ratio, methane fraction, and mixture velocity across a wide range of these variables. Additionally, the use of NTP notably decreases both NO_*X*_ and CO emissions. These findings highlight the potential of using NTP in tandem with CH_4_ addition as a more effective means to stabilize turbulent premixed NH_3_/air flames and reduce harmful emissions, leveraging their synergistic effects.

The research reviewed shows that using nonequilibrium plasma in ammonia combustion effectively enhances the laminar burning velocity by improving the decomposition of ammonia and increasing the equivalence ratio and *E*/*N*, with significant support from hydrogen atoms generated during plasma discharges. Moreover, adjusting the pulse energy density, while keeping *E*/*N* constant, drastically reduces the ignition delay time and significantly increases the burning velocity. The introduction of nonequilibrium plasma also shortens the ignition delay time by generating reactive OH radicals. Furthermore, nonequilibrium plasma broadens the lean blowout limit, enhancing performance marginally better than when hydrogen is used. It also lowers NO_*X*_ emissions, particularly when the discharge power and voltage are increased. Specifically, plasma-assisted ammonia combustion reduces NO_*X*_ formation significantly, primarily by hastening the conversion of NH_3_ to N_2_, thus minimizing the precursors available during ignition that contribute to NO_*X*_ production.

### 2.5 Ammonia Combustion in Internal Combustion Engines

Internal combustion engines are extremely versatile, in terms of both the fuel that can be used and its applications. The earliest successful attempts to use ammonia as a fuel for cars and buses goes back to the 1930s–1940s.^10,111^ In the 1960s, U.S. institutions investigated the potential of ammonia as an alternative fuel using cooperative fuel research (CFR) engines. This led to a set of publications which demonstrated the feasibility, not without challenges, to run ICE on ammonia-based fuel.^112−115^ The interests of the industry and academia only took off in the late 2000s as limits on carbon emissions were becoming more stringent to look for alternatives to hydrocarbon fuels. Major engine manufacturers of the shipping industry such as Wärtsilä and MAN Energy Solutions have announced their first operational ammonia-based engines in 2024.^116,117^

Compared to standard fuels like gasoline and diesel or even hydrogen, most of ammonia’s properties make it hard to burn.^118^ Ammonia possesses a narrow flammability limit, especially compared to hydrogen, and its resistance to autoignition is very high with an autoignition temperature of 651 °C (against 254 °C for diesel and 370 °C for gasoline).^20^ This makes the use of ammonia in a compression ignition (CI) engine very challenging, and very high compression ratios (>35:1) are needed to ignite it.^115^ Therefore, the research mainly approached the use of ammonia in CI engines via dual-fuel operations to compensate for that issue.^119^

The very low flame speed (laminar flame speed is 7 cm/s^120^) slows down the combustion process. Therefore, using a combustion promoter like hydrogen will be key to maintaining a stable combustion and ensure ignition of the mixture in spark ignition (SI) engines. However, ammonia properties are also an advantage regarding knock in SI engines thanks to the very high octane number (>130).^121^ Additionally, the heat of vaporization (1370 kJ/kg)^122^ is very high compared to the range for gasoline (180–350 kJ/kg),^123^ reinforcing knock resistance as spontaneous ignition is unlikely to occur. Although unlikely, knock should not be disregarded especially when considering ammonia fuel blends because optimization solutions like boosting or increase of compression ratio could make it possible.^124^ Another important aspect is the higher fuel consumption when using ammonia. Diesel has a lower heating value of 45 MJ/kg and ammonia only has half that amount with 18.8MJ/kg, meaning that fuel consumption should be expected to be twice that of a standard fossil fuel.^118^

Because of ammonia’s characteristics, its utilization as a fuel in internal combustion engines is challenging. Yet, recent publications proved that it can be achieved with relatively limited changes to the engine itself, which is encouraging, as it shows that retrofit of an existing ICE is possible. The main issue to be addressed in the transition of ICEs toward using ammonia is related to NO_*X*_ emission mitigation while maintaining adequate performance.

#### 2.5.1 SI Engines

The current trend is to favor utilization of ammonia with spark ignition engines to take advantage of the high octane number and because of the reasonable compression ratio thanks to the ignition via a spark plug. However, the slow flame speed of pure ammonia poses problems to maintain stable combustion for a range of operational conditions, and the need for a combustion enhancer has been repeatedly reported in the literature.^112,114,121−123,125,126^ The spectrum of fuels used to promote ammonia combustion includes gasoline, alcohol, methane, natural gas, syngas, and hydrogen.

Overall, an SI engine based on ammonia shows the following trends with respect to combustion enhancement:^112,121,126,127^ At low load, a higher amount of fuel promoter is needed, whereas it is possible to operate on pure ammonia at high loads. Regarding engine speed, at high speed it is difficult if not impossible to maintain stable combustion, and a fuel promoter is needed. The spark timing must be advanced compared with standard fuel operation to allow time for the flame to propagate. Finally, cold start operation requires a higher amount of fuel promoter than once the engine is warmed up.

**Figure 16:**
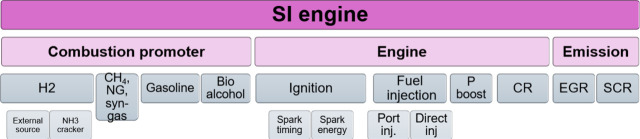
Optimization pathways investigated in the literature for SI engines.

Other strategies for performance improvements (and reduce NO_*X*_ emission) have also been explored such as boosting the intake pressure using a turbo or a supercharger and increasing the compression ratio (CR), which has shown good results.^112,128−131^ The study of different fuel injection strategies either in the intake port (majority of the publications) or by direct injection in the cylinder is also being considered. These strategies are also used in the frame of emission mitigation, where understanding of the ammonia combustion process is essential. Selective catalytic reduction (SCR) is a commonly proposed aftertreatment solution,^129,131,132^ and EGR in combination with rich operation could provide interesting results.^125,126,129^ A summary of all the optimization strategies which are currently investigated is provided in Figure 16.

Gasoline/ammonia blend experiments have shown that a mixture of 30% gasoline and 70% ammonia reached a heat release rate 25% lower than for standard gasoline operation and could provide a similar result if optimized with an intake pressure boosting device.^121,133^ An earlier spark timing was necessary compared with 100% gasoline to ensure stable combustion.

Methane, natural gas,^133,134^ and more recently syngas^128^ have also shown interesting results and are attractive as they can be obtained from sustainable sources. Operation with 50% natural gas was achieved easily, thus reducing by 28% the CO_2_ emissions.^218^ In the case of syngas,^128^ the performance was lower than with pure methane but boosting the intake pressure by 20% then gave similar results. Ammonia slip was relatively important in the exhaust in the case of syngas.

#### 2.5.2 CI Engines

The early studies on compression ignition engines highlighted the difficulty in igniting pure ammonia. The very high compression ratios necessary were not deemed practical and did not provide satisfactory results.^115,135^ Two methods to work around that issue have been proposed: dual fuel (similar to the use of a fuel promoter with SI engines) and the implementation of a spark-assisted operation. The most explored route is the dual-fuel route, used in a small amount to trigger the ignition. Early studies performed in the 1960s tested up to 45 additives (fuel promoter),^114^ and for CRs up to 25:1, acetylene was preferred, whereas above that value hydrogen performed better. Acetylene was also considered less convenient because of the higher amount required. The more recent studies mostly focus on diesel (and biodiesel), dimethyl ether (DME), and hydrogen.^118,120,136,137^

Various combustion modes have been tested covering homogeneous charge compression ignition (HCCI), partially premixed compression ignition (PPCI), and reactivity-controlled compression ignition (RCCI). Stable combustion with HCCI was obtained with a blend of 70% ammonia and 30% H_2_, but efficiency is lower and optimization such as increasing the CR, boosting the intake pressure, and boosting the crevice are needed.^118^ For RCCI, ammonia is used as the low reactivity fuel and the fuel promoter (only diesel has been tested) is used as high reactivity fuel.^136,138^ The diesel pilot injection timing advance plays an important role in reducing emissions of NH_3_ and N_2_O. However, lower brake efficiency has been reported.^138^

The other route of spark-assisted compression ignition (SACI) was first mentioned by Starkman et al.^115^ in 1968, and in 2021, Mounaïm-Rousselle et al.^139^ attempted again to use that method. The results obtained were promising at different engine speeds and low loads and allowed pure ammonia.

**Figure 17:**
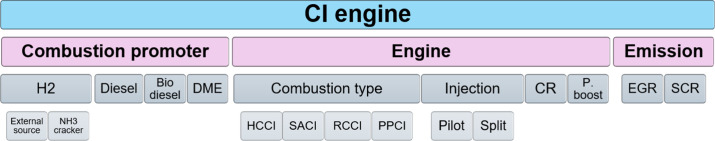
Optimization pathways investigated in the literature for CI engines.

Like for SI engines, emission can be mitigated by using an EGR, but Pochet et al.^140^ reported a negative impact on the combustion efficiency. He suggested to investigate combining EGR with boosted pressure and maximizing the stroke-to-bore ratio to overcome that problem.^28^ SCR is also proposed as a downstream solution to limit emissions. The optimization pathways are summarized in Figure 17.

#### 2.5.3 Emissions Trends for Ammonia Use in ICE

The two major emission contributors are NO_*X*_ (including N_2_O) and ammonia (NH_3_). To a lesser extent, hydrocarbons (HC), carbon monoxide (CO), carbon dioxide (CO_2_), and hydrogen (H_2_) can also be found.

For NO_*X*_, the main trend is that for lean combustion (equivalence ratio ER < 1) more NO_*X*_ are produced, whereas NO_*X*_ production decreases when the operation shifts toward rich combustion (ER > 1);^118,120,138,141^ the opposite behavior is seen for ammonia slip in the exhaust. Overall, NO is in a greater amount but N_2_O is also present, which is critical given its very high global warming potential. Upstream NO_*X*_ reduction methods are being studied via understanding the ammonia combustion characteristics.^138,142^ However, with the current performance obtained, it is expected that SCR will be necessary to keep NO_*X*_ emissions below regulation levels.

The presence of ammonia in the exhaust is attributed to the crevice mechanism suggested by Westlye et al.,^131^ and ammonia slip is more important when operating in rich condition. HC, CO_2_, and CO are also present in the case where the fuel promoter is based on hydrocarbons. Behaviors with emissions vary depending on the engine operation mode. For instance, Niki reported that, for RCCI engine operation, HC, NO_*X*_, and CO increased whereas NH_3_ and N_2_O decreased.^138^ In another publication, Niki suggested that split injection decreased CO and HC.^136^ The emission trends for NO_*X*_ and NH_3_ are presented in Table 1.

**Table 1:** Main Emission Trend Observed for Blend of Ammonia/Other Fuel, Compared to Pure Fossil Fuel from Experimental Results in the Literature^a^

	blend					
	SI engine				CI engine	
emission	H_2_	NH_3_/CH_4_ (NG)	NH_3_/syngas	gasoline	DME	diesel
NH_3_	for increasing ER: stable until ER ∼ 1; increase when ER > 1; decrease with increasing H_2_ content	increase	increase	increase	increase	increase
NO_*X*_	for increasing ER: NO_*X*_ increases until ER ∼ 07.–0.8; NO_*X*_ decrease for richer mixture	increase	slight increase	increase	increase	usually decrease

a Note that the trend may change depending on a particular engine operation type, especially for CI engines.

The main challenge in limiting both NO_*X*_ and ammonia emissions will be to find the sweet spot regarding several aspects that include appropriate fuel blend, injection/ignition method, combustion control, and postcombustion mitigation strategy.

### 2.6 Moderate or Intense Low-Oxygen Dilution (MILD) Combustion

MILD combustion, also termed flameless combustion, has shown a great potential to mitigate the thermal NO_*X*_ problem in NH_3_ combustion. Combustion processes are called MILD when the reactant mixture inlet temperature (*T*_in_) is higher than the mixture’s autoignition temperature (*T*_ign_) and the maximum temperature increase (Δ*T* = *T*_out_ – *T*_in_) after ignition is lower than the *T*_ign_.^143^ The dilution effect can be obtained with effective recirculation, creating a large reaction zone with a relatively uniform temperature field, often leading to limited thermal NO_*X*_ emissions.^144^

#### 2.6.1 MILD Combustion of Pure NH_3_

**Figure 18:**
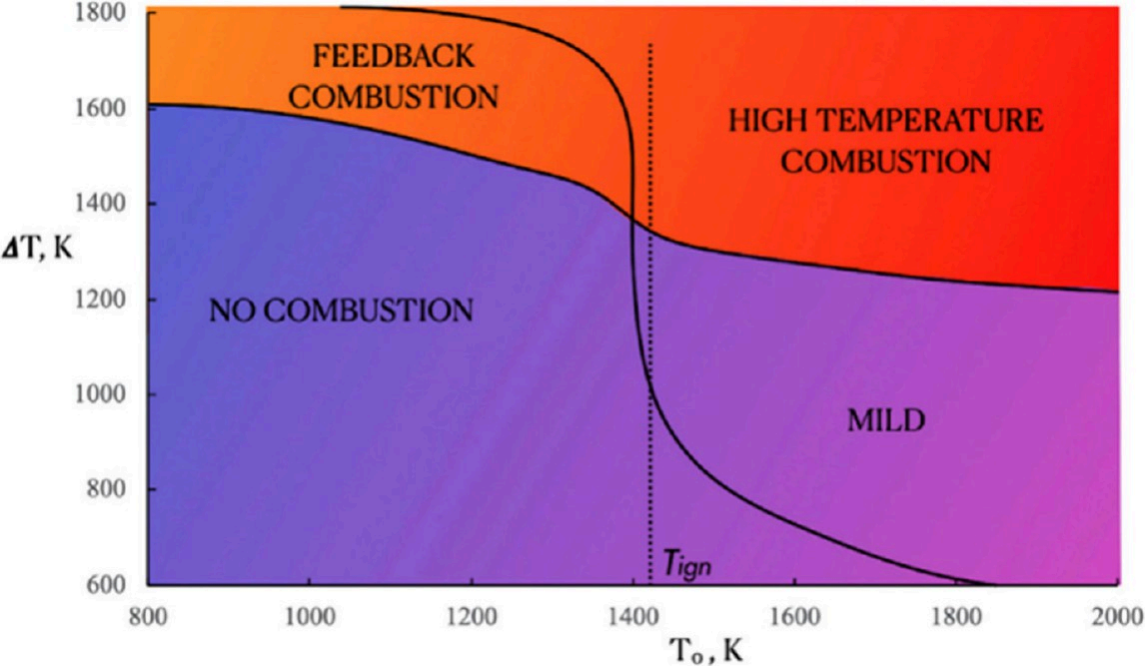
Combustion mode behavior of pure NH_3_ in the hot-fuel–diluted-fuel configuration at *P* = 1 atm. From ref (146). CC BY 4.0.

MILD combustion holds great potential for direct NH_3_ combustion since the oxidation process happens based on the local distributed *T*_ign_ rather than the flame propagation mechanism. Furthermore, it can happen outside the flammability limits due to effective dilution with flow recirculation while achieving reduced thermal NO_*X*_ emissions with moderate reactor temperatures.^145^ These features of MILD combustion offer solutions to low laminar burning velocity, narrow flammability limits, and high thermal NO_*X*_ emission problems of conventional NH_3_ combustion. The MILD combustion mode in the case of pure NH_3_ combustion can be further clarified in Figure 18, with a comparison to traditional combustion modes. The lines separating each combustion mode are computed from heat release and temperature curves.^146^ It is seen that the Δ*T* for the MILD combustion zone is always lower than the *T*_ign_*.*

**Figure 19:**
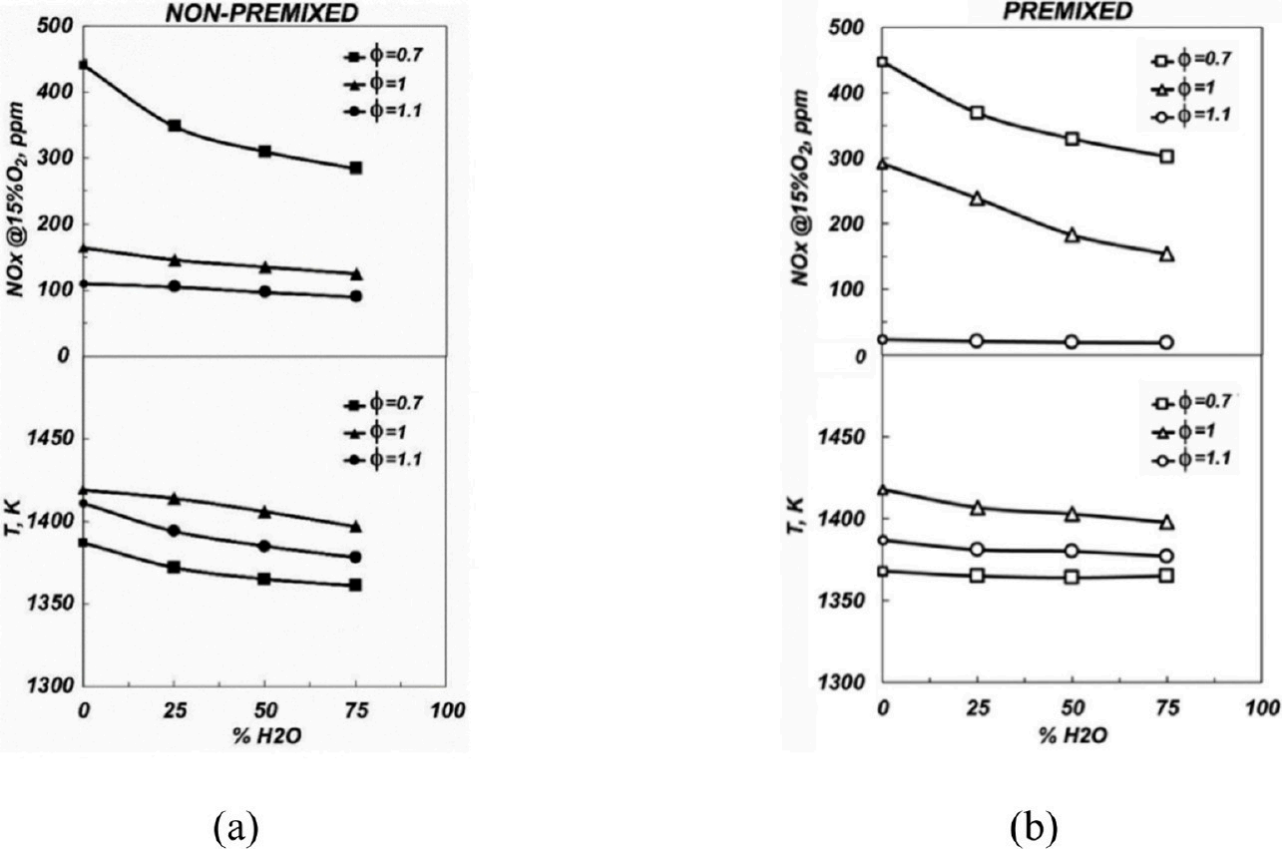
Effect of water addition (% H_2_O) on reactor temperatures (*T*) and NO_*X*_ emissions for varying equivalence ratio at *P*_thermal_ = 7 kW: (a) nonpremixed; (b) premixed. Reproduced with permission from ref (147). Copyright 2021 Elsevier.

The research on the MILD combustion of NH_3_ is still in its early stages and limited. Sorrentino et al.^145^ investigated MILD combustion of pure NH_3_ for the first time using a cyclonic burner configuration. The reactor temperature was kept above 1250 K in all experiments, while stability and emission control were achieved for temperatures higher than 1300 K. The minimum NO_*X*_ levels of lower than 100 ppmv were achieved near stoichiometric conditions (slightly fuel-lean to fuel-rich). It was also noted that unburned NH_3_ content higher than 1000 ppmv was obtained for equivalence ratios above 1.1. Ariemma et al.^147^ studied the effects of water (H_2_O) addition for MILD NH_3_ combustion in a cyclonic burner configuration. It was found that the H_2_O addition reduces NO_*X*_ emissions for both premixed and nonpremixed configurations without compromising process stability. The NO_*X*_ reduction was more pronounced for fuel-lean conditions with increasing H_2_O content, as shown in Figure 19.

Rocha et al.^148^ investigated three NO_*X*_ reduction methods in modern stationary gas turbines, namely, dry-low emissions (DLE), rich-burn, quick-quench, and lean-burn (RQL), and MILD combustion, numerically at a pressure of 20 bar and an inlet temperature of 500 K. The study revealed a strong correlation between NO_*X*_ emissions and the exhaust-gas-recirculation ratio (EGR), indicating a logarithmic decrease in NO_*X*_ emissions with higher EGR levels. It was reported that NO_*X*_ emissions lower than 50 ppmv were possible in highly diluted cases. Recently, Liu et al.^149^ carried out a regime classification study in well-stirred reactor (WSR) combustion. In this study, MILD combustion was classified as a subregime of flameless combustion where NO_*X*_ emissions were lower than 100 ppmv. Furthermore, regarding NO production, the HNO route was found to be the most important producer by contributing more than 94% of the NO in the MILD combustion of NH_3_. Mohammadpour et al.^150^ numerically studied the furnace wall temperature and dilution effects in a furnace. Regarding NO_*X*_ emissions, it was found that the NO_*X*_ emissions reduce significantly when the wall temperatures are reduced and/or the diluent levels in the oxidizer are elevated. Conversely, N_2_O production exhibits an opposing trend, decreasing as the NO_*X*_ levels diminish. Recently, Shi et al.^151^ showed that the MILD NH_3_ combustion reproduces significantly lower NO_*X*_ emissions compared to premixed CH_4_/NH_3_/air combustion due to lower O_2_ concentrations, which subsequently lowers the O, H, and OH radical pool concentrations. Another conclusion was the existence of a critical NH_3_ flow rate under a certain MILD combustion condition. If the critical value is exceeded, then NO_*X*_ emissions increase significantly. Guintini et al.^152^ investigated NH_3_ MILD combustion in a cyclonic reactor configuration at stoichiometric conditions numerically. The work mainly focused on providing a reliable computational framework to reduce uncertainties arising from kinetics, cyclonic flow, and MILD combustion conditions. Regarding the NO_*X*_ emissions, Nakamura et al.^153^ and Li^154^ kinetic mechanisms were found to perform well in terms of relative error compared to experiments. Very recently, Wang et al.^155^ studied the MILD combustion of a premixed NH_3_/air jet flame in hot coflow for varying conditions numerically. Ammonia/air mixtures have wider reaction zones and lower heat release rates resulting in the development of a MILD combustion regime more easily compared to CH_4_/air mixtures. Furthermore, it was seen that MILD combustion reduces the NO_*X*_ emissions of NH_3_/air combustion by 2 orders of magnitude compared to the conventional combustion regime. Also, stoichiometric conditions were found to be the optimal case for NO_*X*_ and unburned NH_3_.

#### 2.6.2 MILD Combustion of Mixtures of NH_3_ with Other Fuels

In traditional combustion, blending NH_3_ with other fuels such as hydrogen (H_2_), methane (CH_4_), syngas, and alcohols is a common practice to enhance certain combustion properties of NH_3_.^156,157^ Lewandowski et al.^158^ explored the NO_*X*_ emissions in MILD combustion of binary NH_3_, H_2_, and CH_4_ blends in a WSR setting. For NH_3_/H_2_ blends, it was concluded that H_2_ content of 10–20 vol % in NH_3_ results in a significant reduction in NO_*X*_ emissions as well as *T*_ign_. For NH_3_/CH_4_ blends, it was seen that NO_*X*_ emissions decrease monotonically as the CH_4_ content increases. Another important finding for NH_3_ blends was the necessity of significant dilution levels, with O_2_ levels around 2–6%, which is necessary to keep NO_*X*_ emissions under 100 ppmv. Similar observations were made in the work of Czyzewski and Slefarski for the co-combustion of ammonia with methane as well as with CH_4_/H_2_/CO_2_ mixture in a semi-industrial chamber for 150 kW burner thermal power.^159,160^ The lowest emissions, not even exceeding 100 ppmv, were obtained under slightly fuel-lean conditions for an equivalence ratio of 0.95 (1% O_2_ in dry exhaust gas). Mousavi et al.^161^ studied MILD combustion of NH_3_/H_2_/CH_4_ ternary mixtures with low NH_3_ concentrations (up to 20 vol %) numerically using a modified eddy dissipation concept (EDC) model. Ammonia addition to the H_2_/CH_4_ mixture resulted in more complete combustion by increasing the flow residence times. Interestingly, addition of NH_3_ increased the reactivity of the mixture unlike the conventional combustion mode. NO emissions were reported to increase sharply near the inlet but then decrease downstream with the addition of NH_3_. Finally, the most important pathway for NO_2_ generation was found to be NO + HO_2_ = NO_2_ + OH. Kuang et al.^162^ investigated the combustion characteristics of various fuel blends containing NH_3_, CH_4_, H_2_, N_2_, and CO_2_ in different combustion modes numerically. The MILD combustion and oxy-fuel MILD combustion modes demonstrated relatively lower NO emissions, specifically for oxy-fuel MILD combustion. Furthermore, the CO_2_ dilution strengthened the MILD combustion state in all scenarios. Similar to their previous work,^162^ Mousavi et al.^163^ used a modified EDC to investigate syngas/NH_3_/CH_4_ mixtures in the MILD combustion regime to investigate stability and NO_*X*_ emissions. It was reported that increasing the syngas content in NH_3_ MILD combustion reduced NO_*X*_ and N*x*O emissions and resulted in more complete combustion. Kiani et al.^164^ experimentally investigated the effect of NH_3_ addition to nonpreheated syngas MILD combustion in a furnace configuration. The mole fraction of NH_3_ was varied between 0.34 and 0.66 at an equivalence ratio of 1.0 for a syngas composition of 0.7; [H_2_]/([H_2_] + [CO]) = 0.7. It was reported that the NO emissions vary between 200 and 400 ppmv and show an increasing trend as the NH_3_ content increases. Later, Jiang et al.^165^ studied syngas/NH_3_ MILD combustion in a novel burner by 20 different experiments. It was reported that low NO_*X*_ emissions could be achieved without any NH_3_ leakage at certain NH_3_ flow rates and furnace temperatures (*T*_furnace_= 1428 K and *V*_NH_3__ ≤ 5.72 SLM, *T*_furnace_ = 1467 K and *V*_NH_3__ ≤ 8.10 SLM). Ariemma et al.^166^ studied the binary NH_3_/alcohol mixtures for methanol, ethanol, and 1-butanol in a cyclonic flow burner. Overall, blending alcohols with NH_3_ provides a stable combustion over a wider range of conditions. The NO_*X*_ emissions were found to be higher than pure NH_3_ but lower than NH_3_/CH_4_ MILD combustion, especially for fuel-lean conditions where NH_3_ slip is lower.

The published literature showed the feasibility of MILD combustion technology in achieving ultralow NO_*X*_ emissions (<100 ppmv). However, several challenging aspects, such as the necessity of high levels of dilution and low thermal energy density, might pose problems in terms of design and operation.

## 3 Insights into Chemical Kinetics Mechanisms for NH_3_-Based Fuels

Over the past six decades, many investigations have been carried out to provide a chemical kinetics mechanism that can interpret the chemical transformation of NH_3_ fuels under gas-phase conditions. These improvements are based on experimental investigations from previous studies, and the results were applied to fit the proposed models. Since the first kinetic reaction mechanism best describing the chemical conversion of NH_3_ oxidation was proposed by Miller et al.,^167^ it has served as the foundation for subsequent efforts to improve kinetic reaction mechanisms.

Despite numerous improvements considering a wide range of operational conditions, equivalence ratios, and mixing ratios in binary flames of NH_3_-based fuels, predictive capabilities still face limitations.^168,169^ This is especially evident in studies focused on NH_3_–H_2_ flames, where prediction accuracy decreases as the hydrogen fraction exceeds 40% in the flame.^49^ The investigation by Girhe et al.^170^ on NH_3_ and NH_3_/H_2_ combustion revealed that the kinetic model proposed by Mei et al.^36^ aligns well with the experimental results of Alzueta et al.,^171^ who conducted flow reactor measurements to study NH_3_/NO interactions. However, the model effectively captures the reduction of NO at the initiation temperature with a decreasing equivalence ratio, a scenario that other tested mechanisms have struggled to replicate. However, the model exhibits poor performance in pyrolysis^170^ and fails to accurately predict the laminar burning velocity (LBV) at high temperatures under fuel-rich conditions, as noted by Shawnam et al.^172^

Establishing a reliable kinetic model that accurately predicts experimental outcomes remains a challenging task. The accuracy of the predictions is fundamentally dependent on the quality and precision of experimental measurements. Limitations in measurement technology have led to a limited understanding of fuel chemistry under combustion conditions, which affects the data used to improve flame kinetics.^49^ Setting aside the kinematic differences among the models, which arise due to the lack of experimental data on the rate coefficients of key reactions, further investigation and adaptation of these coefficients within the kinetic reaction mechanisms are required. Notably, the rate constant of the critical reaction NH_2_ + N_2_O → N_2_H_2_ + NO remains highly uncertain, as reported by Cornell et al.^173^ To address this gap, Wang et al.^174^ recently investigated this reaction under different conditions of NH_3_/N_2_O flames, aiming to refine and update their kinetic model. As a result, chemical kinetics models constantly require refinement and updates to incorporate new experimental observations. Consequently, this section outlines the most widely used reaction mechanisms for NO_*X*_ prediction, highlighting their differences, limitations, and applicability under various conditions and combustion systems.

### 3.1 Nitrogen Oxide Formation Pathways

**Figure 20:**
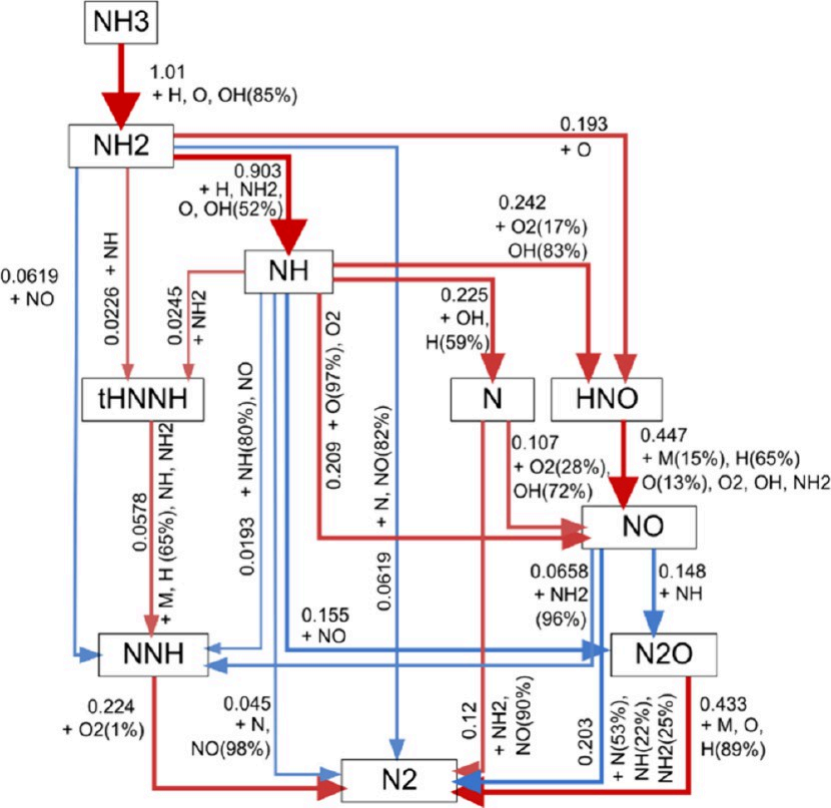
NO pathways at flame temperature (*T* = 1770 K) and at ϕ = 0.8 based on the predictions of the Glarborg kinetic model. Red and blue lines refer to fuel NO_*X*_ pathways and NO reburn pathways, respectively. From ref (175). CC BY 4.0.

The abundance of radicals such as H, OH, and O_2_ significantly influences the combustion environment, promoting the formation of NO through interactions with the ammonia decomposition radicals HNO, NH, and N, which are essential to the pathways leading to NO formation. A study by Alnasif et al.^175^ analyzed the performance of 67 kinetic reaction mechanisms in predicting NO concentrations at various equivalence ratios and atmospheric conditions (Figure 20). The review emphasizes the variation between reaction mechanisms, a description that will not be addressed in this review. Further analyses within such a paper targeted a 70/30 vol % NH_3_/H_2_ premixed flame, highlighting the challenges of using various mechanisms and their resolution processes. The study identified two primary reactions, HNO + H ↔ NO + H_2_ and HNO + OH ↔ NO + H_2_O, as major pathways in the HNO to NO conversion, contributing to increased NO production. Furthermore, the study noted that these reactions display opposite trends as the equivalence ratio (ϕ) increases. Additionally, the reactions N + OH ↔ NO + H and N + O_2_ ↔ NO + O also significantly impact NO formation and are highly dependent on the temperature. Their influence accelerated at higher temperatures, particularly under stoichiometric conditions where temperatures peak, a finding also supported by Hayakawa et al.^54^ in their study on the oxidation of pure ammonia flames. Furthermore, the study by Singh et al.^176^ demonstrated the significance of the reaction NH + O_2_ ↔ HNO + O in increasing NO concentrations, a finding confirmed by Alnasif et al.^177^ study as one of the most influential reactions in NO formation, according to the tested kinetic model of Nakamura.^178^ This reaction produces the important key radical HNO, which serves as a crucial intermediate in NO production.

Increasing the hydrogen content in the binary fuel of NH_3_/H_2_ premixed flames can affect the kinetic chemistry of the NO_*X*_ formation. According to study by Zhang et al.,^179^ increasing hydrogen enrichment accelerates the production of oxygenated radical pools, thereby speeding up NO formation by strengthening the chain branching reaction O_2_ + H ↔ OH + O and the chain propagation reaction OH + H_2_ ↔ H + H_2_O. This leads to an increased generation of H, OH, and O radicals that react with ammonia decomposition products (i.e., NH, NH_2_, NNH, and N) to form NO, a finding also confirmed by Singh et al.^176^

The impact of pressure on the NO_*X*_ concentration has been studied to determine if the chemical reactions related to NO formation in the reaction zone are pressure dependent. According to research by Hayakawa et al.,^54^ increasing pressure significantly accelerates the reaction rates of key reactions such as NH + NO ↔ N_2_O + H, NH + OH ↔ HNO + H, H + O_2_ ↔ O + OH, NH_2_ + O ↔ HNO + H, and OH + H + M ↔ H_2_O + M. Notably, the reaction OH + H + M ↔ H_2_O + M showed the most substantial relative increase. This suggests that OH and H radicals are rapidly consumed in the third-body reaction to form stable H_2_O and play a vital role in NO formation, as emphasized in studies by Miller et al.^167,180^ Consequently, as the pressure increases, the peak NO concentration decreases due to the limited formation of NO, driven by the consumption of OH and H radicals.

When it comes to coal and ammonia cofiring, the reaction mechanism is complex and includes radicals such as NH_*i*_ (*i* = 0, 1, 2), HNO, HNCO, and NCO.^181^ Glarborg et al.^182^ approached the topic, and as depicted later, the group identified the mechanism behind the increase and decrease of NO emissions when both molecules are employed. Further, tar has been acknowledged as a nitrogen carrier in coal. When the temperature is up to 1400 K, tar undergoes rapid pyrolysis to produce HCN.^182^ HCN, as previously described, is an important intermediate of the formation of NO_*X*_, as it can be converted into HNO under the following high temperature reactions.^182^
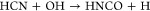
R1
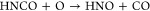
R2

**Figure 21:**
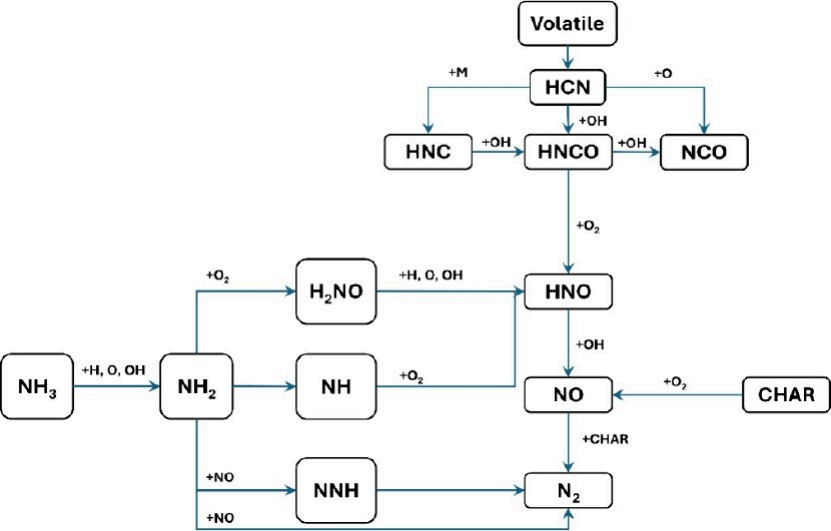
Reaction path of volatiles, ammonia, and char.^183^

Figure 21 shows the dominating reaction paths involved in the conversion of N-containing species in ammonia/coal cofiring with emphasis on the paths followed by HNO. Interestingly, as the ammonia ratio increases, the molecule starts competing for oxygen, thus suppressing HNO. Other NO reactions (R3, R4, and R5) increase until a point where further NH_3_ intake starts De-NO_*X*_ing the combustion system.^182^
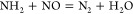
R3
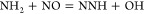
R4
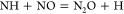
R5

### 3.2 Nitrogen Oxide Consumption Pathways

According to the study of Alnasif et al.,^177^ The key reactions NH_2_ + NO ↔ N_2_ + H_2_O, NH_2_ + NO ↔ NNH + OH, NH + NO ↔ N_2_O + H, NH + NO ↔ N_2_ + OH, and N + NO ↔ N_2_ + O are the most influential reactions in consuming the NO concentration; see Figure 1. The study revealed that the reduction of NO was primarily due to NH_2_, NH, and N radicals, which are accelerated in excess from NH_3_ under rich conditions. This key finding is also confirmed by Glarborg et al.^184^ In scenarios under conditions with 60% hydrogen enrichment, both NH and NH_2_ radicals serve as De-NO_*X*_ agents. They consume NO to form N_2_O and NNH through the reactions NH + NO ↔ N_2_O + H, NH_2_ + NO ↔ NNH + OH, and NH + NO ↔ NNH + O.^184^

N_2_O has been identified in a study by Glarborg et al.^185^ as an important intermediate in the thermal De-NO_*X*_ process. Studies conducted by Miller and Glarborg^186,187^ have identified the reactions NH_2_ + NO_2_ ↔ N_2_O + H_2_O and NH + NO ↔ N_2_O + H as the two main sources of N_2_O production. Similarly, research by Klippenstein et al.^53^ reveals that the formation of N_2_O is significantly influenced by both temperature and oxygen content in the gas mixture. This study demonstrated that the peak concentration of N_2_O shifts from lower to higher temperatures as the concentration of the phosphorus compounds in O_2_ increases.

The consumption of N_2_O primarily occurs through the hydrogen abstraction reaction N_2_O + H ↔ N_2_ + OH, and through dissociation in the presence of a third body (M) via the reaction N_2_O (+M) ↔ N_2_ + O (+M), which is highlighted by the study of Glarborg et al.^185^ The pathways for N_2_O consumption are clearly illustrated in Figure 20, as explained by the study conducted by Alnasif.^175^

### 3.3 Assessment of Kinematic Differences among Reaction Mechanisms

Previous studies have shown that while various kinetic models perform well across different equivalence ratios, some mechanisms only demonstrate good performance with minor discrepancies under specific conditions of ϕ. However, their performance significantly deteriorates when the equivalence ratio either decreases or increases. This apparent good performance might result from coincidental alignments with certain data, rather than accurately reflecting the true kinetic modeling of NO_*X*_ concentration estimation. This indicates a lack of reliability and accuracy in scientific modeling and highlights a deficiency in the performance of ammonia kinetic models.

Alnasif et al.^175^ highlighted in their previous study on burner-stabilized stagnation flames for a 70/30 (vol %) NH_3_/H_2_ mixture that the kinetic model of Nakamura et al.^153^ and the model of Glarborg et al.^55^ perform well from fuel-lean to stoichiometric conditions, showing good agreement with experimental measurements. This suggests that the key reactions influencing the kinetic behavior of NO formation and consumption are effectively captured in the tested mechanisms. However, disparities were observed in the mechanism of Glarborg et al. under conditions nearing ϕ = 1.2, indicating the necessity for enhancements in the rate parameters to more accurately depict the chemical transformation of the relevant reactions for NO.

The study also highlighted the significant roles of key reactions such as HNO + OH ⇌ NO + H_2_O and HNO + H ⇌ NO + H_2_ in NO formation and reactions NH_2_ + NO ⇌ N_2_ + H_2_O, NH + NO ⇌ N_2_ + OH, and NH_2_ + NO ⇌ NNH + OH in NO reduction. Their roles are determined based on the abundance of the radicals H, OH, and O in the flame.^70,176^

According to the analysis conducted by Mulvihill et al.,^188^ the key reaction N_2_O + H_2_ ⇌ N_2_ + H_2_O considerably impacts N_2_O consumption within Zhang’s kinetic model.^189^ However, this reaction is not considered influential in the Klippenstein kinetic model.^190^ Mulvihill et al.^188^ report that the reaction N_2_O + H_2_ ⇌ N_2_ + H_2_O has negligible importance in NO_*X*_ modeling. The study determined that the rate constant for this reaction must be significantly reduced by a factor of 30 from the value used in Zhang’s model. This indicates that the actual rate of the reaction N_2_O + H_2_ ⇌ N_2_ + H_2_O is much lower than previously estimated. The study also revealed that, whether this reaction was completely removed from the model or its rate constant was reduced by a factor of 30, the outcomes were identical. This suggests that the presence or absence of this reaction does not significantly alter the model’s predictions, underscoring that this reaction is not crucial in NO_*X*_ modeling.

The study by Cornell et al.^173^ has brought significant attention to the role of N_2_O in ammonia oxidation at low temperatures. This research involved conducting jet stirred reactor (JSR) experiments using an NH_3_/N_2_O/N_2_ mixture at intermediate temperatures (850–1180 K) and utilized the model of Glarborg^191^ to validate their results and address the data gap concerning N_2_O’s role in the ammonia oxidation process. The study reported that the kinetic model of Glarborg^191^ aligns with the experimental measurements of N_2_O mole fractions when the rate constant of the key reaction N_2_H_2_ + NO ⇌ N_2_O + NH_2_ is reduced by a factor of 10, compared to its original value reported by Dean and Bozzelli.^192^ Furthermore, the study revealed that the predictions of Glarborg’s model, whether including the key reaction N_2_H_2_ + NO ⇌ N_2_O + NH_2_ or not, are nearly identical. This suggests that previous experimental data sets used for validation may not have effectively tested this specific reaction in Glarborg’s model.^191^ As a result, this study underscored the importance of a more accurate determination of the rate constant in future research.

The low-pressure-limit rate constant of the reaction N_2_O + M ⇌ N_2_ + O + M has been thoroughly investigated by Mulvihill et al.^193^ using a shock-tube configuration for mixtures of 0.2% N_2_O/Ar. This study aimed to provide more accurate and reliable data for the reaction N_2_O + M ⇌ N_2_ + O + M in the temperature range 1550–2500 K at 1.3 atm. A detailed kinetic analysis was conducted, accounting for nonideal pressure variations. The study determined that, over the temperature range 1550–2500 K, the rate constant of N_2_O + M ⇌ N_2_ + O + M was best fit by the expression *k* = 1.01 × 10^15^ exp(−30050/*T*), with *k* in cm^3^ mol^–1^ s^–1^ and *T* in kelvin. By integrating these results with previous low-temperature data measured in flow/static reactors, the best fit over the temperature range 850–2500 K was found to be *k* = (1.04 ± 0.04) × 10^15^ exp((−30098 ± 90)/*T*).

The key findings of Mulvihill et al.^193^ differ from those included by the kinetic models,^153,190,194^ which are based on the review studies by Baulch et al.,^195^ and from Zhang’s kinetic model^189^ that is derived from the experimental studies of Javoy et al.^196^ The study’s findings highlight that the concentration of N_2_O is significantly influenced by the key reaction N_2_O + M ⇌ N_2_ + O + M, suggesting that this reaction plays a major role in determining the N_2_O concentration under specific conditions. Therefore, considering these key findings, updating the rate parameters and incorporating the results from the tested conditions, along with low-temperature data from previous studies, will enhance the prediction accuracy of the kinetic models.

### 3.4 Recently Improved Kinetic Models for Nitrogen Oxide Chemistry

Numerous kinetic reaction mechanisms considering the chemical kinetics of NO_*X*_ have been introduced. These mechanisms vary in their mechanistic and kinematic aspects, leading to differences in their predictive outcomes, even though their rate parameters are continuously updated. One of the primary issues that can constrain the kinetic model in certain conditions is the development of the model focused on a specific combustion system under specific operational conditions. Restricting the kinetic model to these conditions can impact its predictive performance, particularly in terms of alignment with experimental observations.

A kinetic model for nitrogen chemistry in combustion was developed by Glarborg et al.,^191^ based on previous work,^53,184,197^ and validated using experimental data from the literature. This model accurately predicts NO formation and consumption across a broad range of conditions. It was later refined by the same author,^198^ maintaining the rate coefficients and thermodynamic data from the original model.^191^ Enhancements included updating the NH_3_/NO_2_/O_2_ reaction subset based on experimental observations from batch reactors (580–690 K) and flow reactors (850–1350 K) within the NH_3_/NO_2_ system. These updates were supplemented with new flow reactor results, examining the impact of O_2_ addition at the same temperatures. The findings are crucial for modeling ignition and N_2_O emissions in ammonia combustion. The results align with the theoretical study by Klippenstein et al.^199^ and are consistent with low-temperature measurements by Lindholm and Hershberger^200^ and Sun et al.^201^

Zhang et al.^179^ developed a model based on the recent study by Mei et al.,^45^ adopting the hydrogen mechanism from Glarborg’s studies,^202^ which was based on comprehensive experimental data on hydrogen combustion. This model incorporates several chemical reactions (H + O_2_ + H/O/OH = products), additions not originally included in Glarborg’s hydrogen mechanism. The base model was validated using experimental data from a jet-stirred reactor (JSR) that tested NH_3_/H_2_ mixtures at atmospheric pressure, with hydrogen content ranging from 0 to 70 vol %. The experimental conditions covered temperatures from 800 to 1280 K and equivalence ratios of 0.25 and 1. Speciation data for NH_3_, H_2_O, NO, and N_2_O were utilized to verify the reliability of the submechanisms for NO and N_2_O, which are crucial intermediates in ammonia oxidation. The key findings of their study indicate that increasing the H_2_ content causes the reaction NH_3_ + H ↔ NH_2_ + H_2_ to proceed in the reverse direction, converting the NH_2_ radical back into an H atom. These newly formed H atoms then initiate further reactions, specifically H + O_2_ ↔ O + OH and H + O_2_ (+M) ↔ HO_2_ (+M), which produce O, OH, and HO_2_ radicals. Additionally, NH_2_ and H_2_NO radicals serve as chain carriers, transforming one HO_2_ and one H radical into two OH radicals, thereby enhancing OH production. This process promotes the consumption of ammonia (NH_3_) and influences the NO_*X*_ formation.

The study by Singh et al.^176^ introduces a new kinetic reaction model for NH_3_/H_2_/air flames based on the kinetic model previously developed by Shrestha et al.^169^ This model takes into account the chemistry of NH_2_, H_2_NO, HO_2_, N_2_H_*X*_, NNH, HNO, and NO_*X*_ emissions in a hydrogen-enriched environment and their effects on the overall pathways. Additionally, the study considers reactions of NO and NO_2_ with NH_2_ radicals due to their impact on the ammonia oxidation process. Their study underscores the significant impact of the chain branching reaction O_2_ + H → OH + O on the overall chemistry, greatly affecting NH_3_/H_2_/air oxidation and emissions. It also highlights the role of HNO in NO production, especially through the reaction NH + O_2_ → HNO + O, which is critical for NO formation. Furthermore, the study found that, at higher levels of hydrogen enrichment, reactions involving NH_2_, NH, NNH, and HNO subspecies also markedly influence the oxidation and NO_*X*_ chemistry in both pure ammonia and hydrogen-enriched ammonia flames.

According to the study of Meng et al.,^203^ which introduced a novel potential NO_*X*_ formation mechanism involving the breaking of the N–N bond through reactions with HNNO, the research collectively refers to *trans*-HNNO, *cis*-HNNO, and ONHN isomers of HNNO. These HNNO forms are produced through the pressure-dependent reaction H + N_2_O (+M) ⇌ HNNO (+M). They engage in various reactions with common combustion species, leading to NO_*X*_ production. The study highlights that at lower temperatures and higher pressures HNNO becomes the preferred product of the H + N_2_O reaction. The study also investigated HNNO interactions with common combustion species like O_2_ and clarified that, while these reactions mainly convert HNNO back to N_2_O, they are relatively slow. This slowness makes interactions with radical species more probable, resulting in high NO_*X*_ yields.

In line with Meng et al.^203^ study, Lee et al.^204^ research confirmed the significant role of the HNNO mechanism in NO_*X*_ formation at high pressures and low temperatures. This confirmation was based on experimental measurements involving H_2_, O_2_, N_2_, N_2_O, NO, and Ar in jet stirred reactor experiments, which provided strong experimental support for the NO_*X*_ formation route via HNNO. The study compared these measurements with highly validated kinetic models^53,70,189,191,205^ for the N_2_O, H_2_, H_2_O, and O_2_ species mole fraction measurements. While the tested kinetic models varied in predicting the onset temperature for reactivity, they generally reproduced the qualitative features of temperature-dependent reactivity. However, for NO, NO_*X*_, and NH_3_, none of the models captured the observed behavior, predicting negligible amounts of NO and NO_*X*_ at lower temperatures (below ∼950 K). In contrast, the model incorporating HNNO pathways predicted significant NO and NH_3_ formation at these lower temperatures (∼800–950 K), unlike models without HNNO. Therefore, including the HNNO pathway in NO_*X*_ formation models marks a significant improvement in accurately predicting and understanding NO_*X*_ emissions in combustion systems.

## 4 Reduced Chemistry for Ammonia System Based on Low-Dimensional Manifold Concept

The chemical kinetics of the ammonia/air system, enriched with hydrogen or methane, involves over 30 species reacting in several hundreds to thousands of reactions. Utilizing detailed chemical kinetics for turbulent flame simulations results in high computational costs due to the high dimensionality and stiffness of the governing equations for species.^206^ Consequently, efforts have been focused on reducing the computational time by developing model reduction techniques for chemical kinetics. Various approaches include the intrinsic low-dimensional manifold (ILDM),^207^ computational singular perturbation (CSP),^208^ global quasi-linearization (GQL),^209^ reaction–diffusion manifold (REDIM),^210^ flamelet-generated manifold (FGM),^211^ flamelet/progress variable (FPV),^212^ and others (refer to ref (206)).

In ref (213), the CSP method was utilized to identify fast species that can be considered to be in quasi-steady state. For instance, the CSP tool determined that NH_2_ and HO_2_ reach a quasi-steady state. In ref (214), the CSP method was applied to a perfectly stirred reactor (PSR) for a premixed ammonia/air system. Analysis of chemical time scales revealed NH and H_2_O as two species with short chemical time scales for the PSR model, making them prime candidates for steady-state species. Further CSP analysis demonstrated that the chemical time scales of OH and NH_2_ radicals are 2 orders of magnitude shorter than those of major species such as N_2_O, N_2_, O_2_, and H_2_.

The chemical kinetics were further analyzed using the GQL method, which is capable of globally studying fast and slow chemical processes. In ref (215), the GQL reduction method was applied to the CRECK mechanism, comprising 203 elementary reactions and 30 species. It was demonstrated that, for reproducing ignition delay times with high accuracy (<5% error), a 17-dimensional GQL reduced chemistry is adequate. Additionally, to capture the low-temperature reaction of ammonia with NO_*X*_, a 16-dimensional GQL reduced chemistry is necessary.

In turbulent reacting flows, reduced chemistry based on the low-dimensional manifold concept offers computational efficiency, typically involving only two or three progress variables. The flamelet model shows a balance between accuracy and computational cost, making it a practical choice for simulating turbulent combustion across various engineering applications. For instance, the FGM model has been integrated with LES^68^ or RANS^216^ to simulate swirling flames in NH_3_/CH_4_/air systems. One of the most challenging aspects of the flamelet model is defining progress variables (PVs), which are used to parametrize the thermokinetic states. Various definitions for different combustion configurations are listed in Table 2.

**Table 2:** Definitions for Different Combustion Configurations

	PV	test case	ref
CH_4_/NH_3_/air	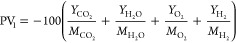	swirling flames	(147)
	PV_2_ = enthalpy		
CH_4_/NH_3_/air	PV_1_ = *Y*_CO_2__ + *Y*_CO_	swirling flames	(216)
	PV_2_ = enthalpy		
coal NH_3_	PV = *Y*_CO_2__ + *Y*_CO_ + *Y*_H_2__ + *Y*_H_2_O_	cofiring of ammonia with pulverized coal	(217)
coal NH_3_	PV = *Y*_CO_2__ + *Y*_H_2__ + *Y*_H_2_O_	laminar counterflow diffusion flames of pulverized coals	(218)
NH_3_/air	PV_1_ = *Y*_N_2__ + *Y*_H_2__ + *Y*_H_2_O_	laminar premixed flame	(219)
	PV_2_ = mixture fraction		
NH_3_/H_2_/air	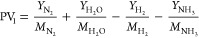	turbulent cracked ammonia flame	(220)
	PV_2_ = enthalpy		

The choice of suitable progress variables aims at predicting the NO_*X*_ emissions accurately. In most cases listed above, the transport equations for NO_*X*_ species must be considered due to their related slow chemical reactions. It was found in ref (72) that the prediction of NO_*X*_ emissions can be improved by considering the NO transport equation in the simulation. However, if one considers the multidimensional cylindrical ammonia/hydrogen flames in which flame stretch exists, as studied in ref (219), the preferential effect has significantly influenced NO_*X*_ emission, and the other selection of progress variables must be used to capture the NO_*X*_ concentration. To summarize, for an accurate prediction of NO_*X*_ emissions, a great deal of attention must be paid to the selection of suitable progress variables in the flamelet model for the turbulent ammonia flame simulation.

## 5 CFD Modeling: An Overview

In computational fluid dynamics, reactive simulations play a crucial role in understanding the formation of NO_*X*_ from ammonia combustion. Ammonia chemistry proves to be more challenging than that of fuels commonly utilized in gas turbines,^10,221^ such as natural gas and hydrogen, especially in terms of comprehensively resolving mechanisms related to NO_*X*_ emissions, as previously depicted. To improve the combustor design procedures by investigating the complexities of pollutant emission formation paths, a proper definition of the ammonia combustion mechanisms, combined with various turbulence closures like Reynolds-averaged Navier–Stokes (RANS), large eddy simulation (LES), and direct numerical simulation (DNS) is required. RANS approaches have been widely used as a standard predictive tool for combustion applications at the industrial level. Even though they remain an attractive choice for providing quick indications and trends, reliable predictions of pollutant emissions require the adoption of detailed chemistries integrated with an efficient turbulence–chemistry interaction model obtained by performing detailed LES. Conversely, at the state of the art, DNS is totally unfeasible for NO_*X*_ prediction along industrial geometries due to the formidable computational costs. Nevertheless, its application to academic cases is essential to shed some light on the complex mechanism of the fuel bound formation.

**Figure 22:**
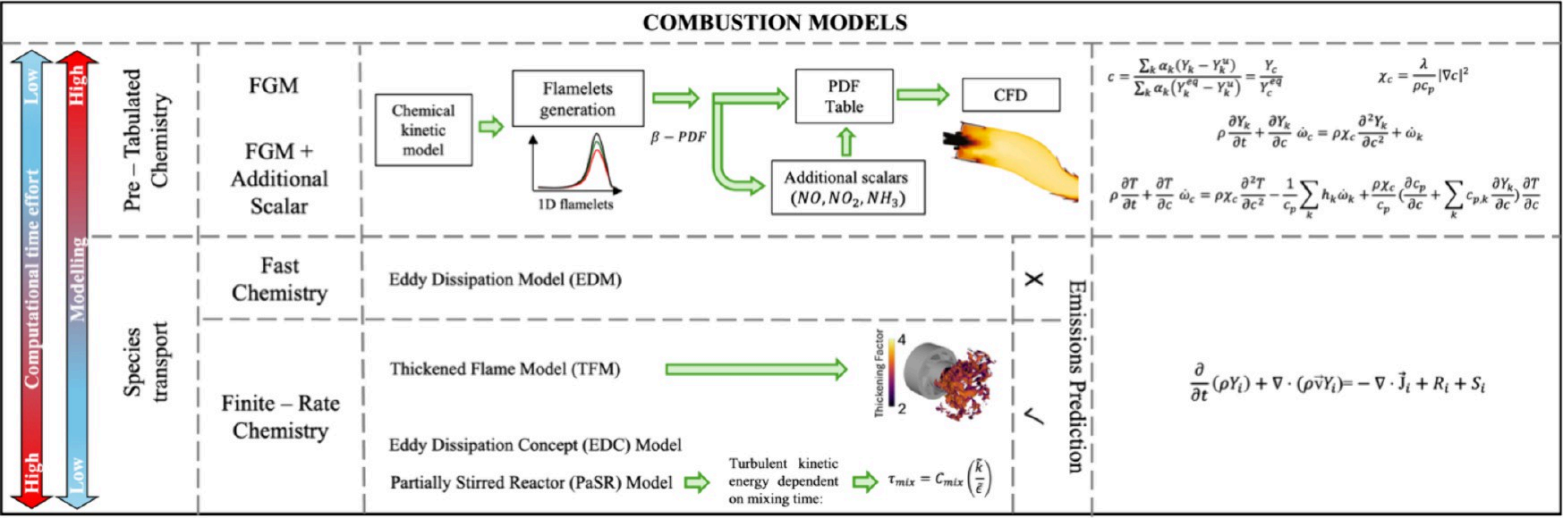
Combustion models comparison in terms of computational time effort and modeling.

The complexity and the computational cost of the simulation are primarily determined by the selected combustion model; thus, it is essential to distinguish between two applicable approaches in reactive CFD, as reported in Figure 22. Models based on the transport of the primitive variables (so-called species transport) resolve the combustion using a chemical mechanism that could account for several chemical species and hundreds/thousands of reactions. Such an approach can provide highly accurate predictions by tracking individual chemical species within the computational domain. However, this approach is computationally expensive due to the need to solve many coupled differential equations, especially in the LES context. The adoption of such a solution strategy along industrial geometries is very hard to implement, unless skeletal chemistries derived from detailed chemistry sets are used.

In the species transport category, the eddy dissipation concept (EDC) and partially stirred reactor (PaSR) combustion models should be mentioned. The former, proposed by Magnussen,^222^ is a finite-rate chemistry combustion model, widely applied for the simulation of combustion systems, although it requires not negligible computational costs. The PaSR model, proposed by Chomiak et al.,^223^ represents a perfectly stirred reactor (PSR) with imperfect mixing. Like EDC, PaSR conceptualizes combustion as a series of reaction and mixing steps within locally uniform regions with the definition of a reacting volume fraction based on local estimations of chemical and mixing time scales.

On the other side, pretabulated chemistry approaches like flamelet-generated manifold (FGM)^211^ represent a good trade-off between accuracy and computational efficiency. In fact, the combustion is modeled through a low-dimensional manifold derived from the chemical kinetics while the turbulence–chemistry interaction is usually calculated through a β-PDF function of auxiliary variables like mixture fraction and progress variable.^224^ By precomputing 1D flamelets, prechemistry significantly reduces the computational costs. However, it may not capture all of the combustion features that are accessible when solving the species transport approach. As an example, computation of the characteristic chemical time scale is generally one of the variables that is used to define the progress variable. By using FGM it is not possible to capture the trends of those species, like NO_*X*_, whose formation time scale is quite different from the other, much faster, species. As it will be pointed out in the literature review, pretabulated approaches struggle in the NO_*X*_ prediction even when coupled with high-fidelity turbulence models like LES.^80^

Recently, a new approach combining CFD and chemical reactor networks (CRNs)^225^ has received significant attention, with the goal of reducing simulation time and computational effort. CRN represents a hybrid approach that combines zero-dimensional perfectly stirred reactors (PSRs) with one-dimensional plug-flow reactors (PFRs). Ehrhardt et al.^226^ were the first to introduce this approach, which consists of three main steps: (a) A reactive CFD simulation is performed, possibly limiting as much as possible the computational effort, for example using global kinetic scheme. The CFD results are then postprocessed using a set of global criteria to separate the combustor into chemically and physically homogeneous zones. The cells that satisfy the same criteria are clustered together to form the zones of the reactor network. (b) A perfectly stirred reactor (PSR) or a plug-flow reactor (PFR) is associated with each zone according to the local flow conditions. The links and the exchanges of the main physical quantities between the reactors are established by computing the mass fluxes between adjacent zones. (c) Last, the CRN is solved with a detailed chemical reaction mechanism to obtain an accurate prediction of pollutant emissions. The CRN model is significantly less computationally intensive compared to the above-described approaches, and therefore, it can efficiently be used to perform many simulations, comparing several chemical kinetics models. Obviously, the reliability of the CRN predictions is strongly dependent on the accuracy of the underlying CFD simulation that in any case must be able to capture the main features of the flame, such as its morphology, length, and position inside the combustor.

In the next section, a comprehensive review will report the main works present in the literature employing the approaches here described, aiming at highlighting the main findings as well as their limitations.

### 5.1 Comprehensive Review on NO_*X*_ Emission Evaluation by CFD

**Figure 23:**
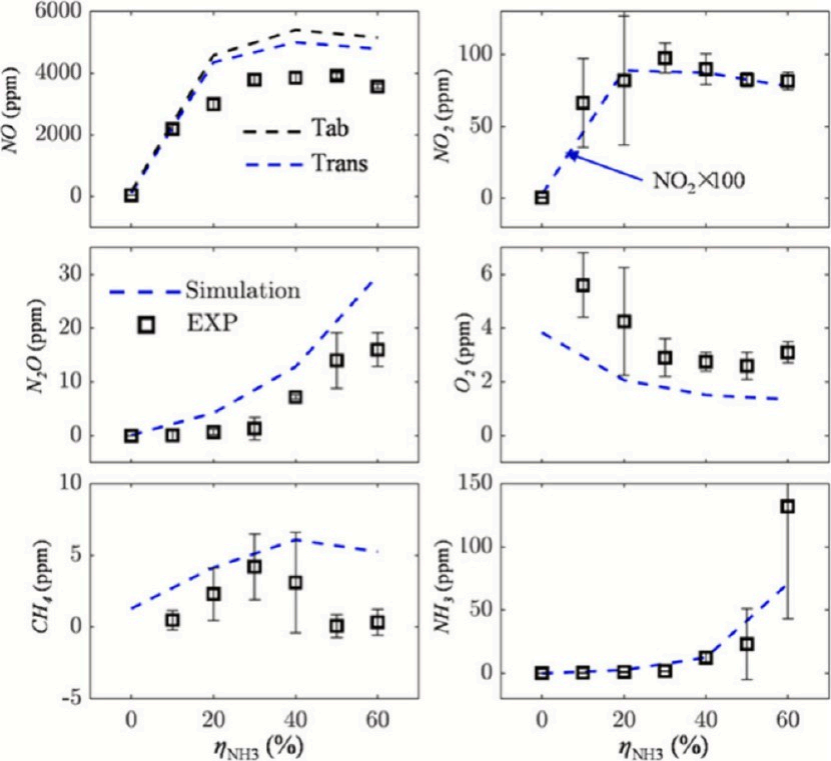
In figure of NO (ppm), comparison of NO transported values and pretabulated values with experiments. Comparison of experimentally and numerically derived values of NO, NO_2_, N_2_O, O_2_ and unburnt fuel (CH_4_ and NH_3_) emissions as a function of NH3 fraction. Reproduced with permission from ref (68). Copyright 2021 Elsevier.

As for the pretabulated chemistry models, an interesting solution strategy to overcome the issue related to the different time scales between NO_*X*_ and the species used for the definition of the progress variable is represented by the solution of additional scalar transport equations associated with NO_*X*_ whose source terms are pretabulated as well, with the advantage to decouple the NO_*X*_ equation from the remaining set of transport equations. This approach has been adopted by Cerutti et al.,^227^ where an initial feasibility study on introducing ammonia into industrial gas turbines by combining LES and FGM combustion models and NO_*X*_ emissions was investigated. It was confirmed that fuel-bound NO_*X*_ plays a dominant role in pollutant emissions. The simulations captured the key physical trends, revealing appropriate tools for assessing the feasibility of introducing hydrogen–ammonia mixtures in gas turbine combustors. An et al.^68^ employ such method for modeling the FGM combustion in a fully premixed swirled flame investigated through an LES: the research is focused on the impact of different NH_3_ concentrations blended with CH_4_. While excellent agreement with the data is retrieved in terms of flame position and velocity field, the prediction of NO_*X*_ is not fully satisfactory. Despite a significant improvement of the results obtained through the decoupling approach if compared to the pretabulated NO mass fraction, as shown in Figure 23, the model results in a marginal error only when low percentages of NH_3_ (by volume) are present in the mixture. Conversely, when the fuel-bound NO_*X*_ pathways are preponderant over the thermal pathways, the error increases significantly. A huge overprediction of the emission is noticeable, as the numerical model predicts NO_*X*_ values 1000 ppm higher than the reference data. The same numerical approach is employed by Wang et al.^228^ focusing on a nonpremixed flame. The fuel gas is obtained by ammonia cracking, and two different concentrations of NH_3_ are investigated, namely 56 and 75%. Detailed radial NO profiles measured at several axial distances from the injector are used to measure the performance of the LES employing Okafor’s mechanism.^42^ Interestingly, there is a very good match in the sections close to the injector, while the prediction degrades moving downstream. It can be seen that the LES overpredicts the data by about 50% at the last reported cross section. Honzawa et al.^229^ focus their investigation through LES on the effect of heat losses on NO_*X*_ production in a perfectly premixed test rig including this effect in the FGM model. The LES simulations are performed for a NH_3_–CH_4_ blend, providing an assessment also for unburnt CO. Despite a significant improvement of NO_*X*_ prediction obtained through the nonadiabatic model, demonstrating the sensitivity of the fuel-bound NO_*X*_ to the heat loss, the difference with the reference data is still significant: while in the rich regime the model overpredicts the emission with a factor of 1.5, in the lean regime NO_*X*_ are up to 5 times the experimental value. An interesting finding coming from this study is that all of the chemical mechanisms that have been tested behave in a similar way.

A different approach was attempted by Yadav et al.,^217^ conducting LES modeling of cofiring of ammonia with pulverized coal in a single-burner test furnace and studying the nonadiabatic effects using a three mixture fractions flamelet/progress variable (3Z-FPV) methodology. A six-variables tabulation method based on nonpremixed flamelets was introduced. To parametrize the thermochemical space, the model used three mixture fractions—ammonia, coal volatiles, and char off-gases—as well as the variances of mixture fraction, reaction progress variable, and total enthalpy. A direct comparison between the adiabatic and the nonadiabatic models was shown and compared with the available experimental data. The results demonstrate that the NO emissions and temperature are slightly underestimated in the nonadiabatic case, while an opposite situation (overprediction) is noticed in the adiabatic case. Aiming at obtaining hydrogen from the cracking process, two ammonia cracking ratios of 14 and 28% at a pressure of 5 bar were examined by Wang et al.^228^ The authors explored the effect of the ammonia cracking ratio on the flame structure and NO formation mechanism. The chemical kinetics schemes of Otomo et al.,^70^ Okafor et al.,^42^ Mathieu et al.,^230^ and Jiang et al.^231^ were compared to determine the best solution for replicating these operating conditions. The FGM approach was employed in the context of large eddy simulations, and the results were compared with experimental data available in ref (232). It was shown that with increasing cracking ratio the flame reactivity is enhanced but also the generation of NO is increased. Good correlations between numerical simulation and experimental data have been obtained, particularly in the flame zone, while the error becomes not negligible downstream from the fuel inlet section.

The higher accuracy of the species transport models over the pretabulated ones is demonstrated by Meloni et al.,^233^ by investigating the effect of rising operating pressure along an extremely lean NH_3_–H_2_ blend. Here, a perfect match of the NO emission is obtained at atmospheric pressure, while a 15% lower concentration is obtained at the maximum operating pressure of 2 bar. Bioche et al.^234^ use LES to predict the flame characteristics and the NO_*X*_ emissions using the PRECCINSTA burner. A dynamic Smagorinsky and TFLES models are used for the subgrid scale and the turbulent combustion models, respectively. They found an emission minimum point at a rich condition (ϕ = 1.46) for *X*_H_2__ = 0.46, corresponding to *X*_NO_*X*__ ≈ *X*_NH_3__ ≈ 300 ppmv. The same computational domain (PRECCINSTA) was used by Shen et al.^235^ employing CFD simulations to investigate wet ammonia combustion characteristics and the NO_*X*_ formation mechanisms using LES. The effects of the equivalence ratio and the amount of hydrogen in the mixture have been analyzed in terms of fluid dynamics variables and emissions. The partially stirred reactor (PaSR) is used as a combustion model with finite-rate chemistry. A sensitivity analysis showed the influence of various parameters on NO_*X*_ emissions, velocity, and temperature fields, providing valuable information for optimizing wet ammonia combustion systems. However, the lack of validation against experimental data limits the reliability of the findings. As for the flame morphology, the wet combustion leads to the liftoff of the flame, while an attached flame to the bluff body is identified for the dry flame. The rich condition shows the superiority of controlling NO and N_2_O emissions via the reburning of NO downstream. However, the unburnt ammonia remains at problematic levels due to the limited residence time in the combustion chamber. Finally, a POD analysis was carried out to extract the coherent structures and ascertain the nonlinear turbulence–chemistry coupling mechanism. Indlekofer et al.^236^ carried out a comprehensive study that exploits the use of large eddy simulations in conjunction with detailed chemical kinetics to assess a rich–lean staging strategy applied to the combustion of partially cracked ammonia. A CRN model is developed and tuned against the LES results with the goal to gain more detailed insights about the chemical pathways. The analysis of the results obtained from both numerical modeling approaches, LES and CRN, confirmed the experimental findings and predicted a significant reduction in NO_*X*_ emissions compared to the nonstaged case. The two-staged combustion is also investigated by Okafor et al.^66^ for methane–ammonia–air diffusive flames using LES coupled with the PaSR combustion model; the optimum equivalence ratio in primary zone was found to vary from 1.30 to 1.35 as the ammonia fuel fraction decreased from 0.30 to 0.10.

Moving to methodologies requiring lower computational effort, Füzesi et al.^237^ employed a RANS turbulence model coupled with a partially stirred reactor for the analysis of a lean premixed swirl-stabilized burner operating at atmospheric pressure. The analysis involves a blend of NH_3_–H_2_ at several equivalence ratios (up to 0.9) and H_2_ shares (up to 20 vol %). The comparison with data at 10% H_2_ reveals that the trend of NO (predicted through Okafor’s mechanism^42^) is satisfactorily predicted. It was shown that NO rises with the equivalence ratio, and a mean error of about 15% was measured across the investigated conditions. Mikulčić et al.^238^ and Chaturvedi et al.^225^ performed atmospheric RANS simulations with a finite-rate combustion model for premixed NH_3_–CH_4_ and NH_3_–H_2_ blends, respectively. In both cases, the CFD is coupled with a chemical reactor network investigating several chemical mechanisms. In the former work, three of the most common chemistry sets used in the literature for ammonia combustion are analyzed. All the mechanisms showed a satisfying agreement with the reference data only for ultrarich conditions, while a huge overprediction can be identified in lean conditions for any employed chemistry set. Furthermore, a non-negligible difference can be observed among the mechanisms under the same operating condition. Very similar trends are obtained in Chaturvedi et al.,^225^ with a significant overpredictions of the data at lean conditions for all the investigated chemistry sets. Such a gap is progressively reduced moving close to the stoichiometric and rich conditions. Mazzotta et al.^239^ used RANS simulations coupled with detailed chemistry, specifically EDC and PaSR combustion models, to compare the effects of pressure on NO_*X*_ emissions derived from ammonia–hydrogen blends as a results of high ammonia cracking. In particular, a mixture consisting in 25% NH_3_–75% H_2_ (by volume) was analyzed. The increase in pressure from 0.11 to 0.2 MPa resulted in a reduction of 65% in NO_*X*_ emissions. A comparison of various kinetic schemes in laminar burning velocity, ignition delay time, and emissions was carried out, aiming at selecting the most suitable one to be used in subsequent CFD simulations. The choice of chemical kinetics model resulted to be a key parameter in terms of prediction of combustion and emission characteristics. However, even if the numerical results derived by both EDC and PaSR predicted the experimental NO_*X*_ emissions with small errors, the computational time turned out to be prohibitive due to the combustion model being based on detailed chemistry to obtain a narrow range of results. A similar approach to the latter was analyzed by Vigueras-Zuniga et al.^240^ using RANS simulations coupled with the EDC model on hydrogen–ammonia mixtures. The model demonstrated good accuracy in the prediction of the consumption and production of ammonia under atmospheric conditions. However, the results confirmed the poor capabilities in the prediction of nitrogen oxide emissions and hydrogen reactivity with an overestimation of both parameters. The effect of pressure is investigated by Somarathne et al.^241^ for premixed ammonia–air flames: the increase in pressure from 0.1 to 0.5 MPa results in a reduction of NO from 700 to 200 ppm. Sun et al.^242^ employed RANS coupled with the EDC combustion model to analyze the combustion and emission characteristics of premixed ammonia and hydrogen flames in a swirling flow configuration. The effects of the equivalence ratio and hydrogen content in the mixture for NO emissions were investigated. The increase in the equivalence ratio leads a decrease in NO emissions for ϕ > 1.1, while unburned NH_3_ increases in rich condition, at ϕ > 1.3. As the hydrogen content is increased, unburnt NH_3_ is decreased dramatically, while NO generation is increased. As *X*_H_2__ is increased, NO emission is increased significantly at a lower ϕ, while it is increased slightly or even remains unchanged at a higher ϕ.

The formation and distribution of nitrogen oxides (NO_*X*_), including NO, NO_2_, and N_2_O, are crucial due to their environmental impact, and CFD has become an indispensable tool for predicting and analyzing the distribution of these species within the flame structure and the overall combustion device to try to identify and reduce the most impactful NO_*X*_-forming reactions. Direct numerical simulation (DNS) offers a unique capability to quantify these radicals with high precision, capturing the intricate interactions between turbulence and chemical reactions. In particular, DNS provides a detailed spatial distribution of NO, NO_2_, and N_2_O within the flame front, allowing for the identification of regions with high pollutant formation. In contrast, large eddy simulation (LES), while effective for larger scales, lacks the resolution needed to accurately capture these finer-scale species distributions. However, the introduction of hydrogen into an ammonia-based flame front introduces a greater degree of uncertainty about the formation of NO, NO_2_, and N_2_O. This uncertainty is primarily due to the difficulty in modeling mixtures with *Le* ≠ 1, necessitating a deeper analysis of preferential and differential diffusion.^219,243^ Tian et al.^244^ analyzed turbulent ammonia/air premixed flames to investigate the influence of the equivalence ratio on the flame structure and NO formation characteristics using DNS and analyzing the role of preferential diffusion. It was found that NO is high in the product of the lean case, as well as NO_2_, even if it is 3 times smaller. In contrast, NH_3_ is higher in the product of the rich case. The effect of molecular diffusion on the turbulent premixed NH_3_/H_2_/air flame structure and NO production were investigated by Chi et al.^245^ by performing four different DNSs, highlighting the need to include the molecular diffusion and the Soret effect to better capture the flame structure, in particular in the prediction of the NO production and turbulent flame speed. Osipova et al.^246^ investigated experimentally and numerically the flame structure of premixed ammonia/hydrogen flames at 4 and 6 atm while varying the equivalence ratio between 0.8 and 1.2. According to experimental data and numerical simulations, NO was the most abundant nitrogen-containing species in the postflame zone, while N_2_O and NO_2_ concentrations were negligible. Transitioning to rich conditions (ϕ = 1.2) reduced NO concentrations in the postflame zone as well as peak NO and N_2_O concentrations in the reaction zone.

### 5.2 Final Considerations

To date, the prediction of NO_*X*_ emissions in mixtures with ammonia via CFD is still an inaccurate and challenging topic, especially due to the difficult prediction of NO and NO_2_ formation from fuel-bound NO_*X*_ mechanisms. The combustion models that are most effective, and within a feasible error range, are those that couple detailed chemistry using species transport models (EDC or PaSR) with unsteady simulations (LES) to predict the formation of slow chemical species; however, the computational effort required is prohibitive. Models based on pretabulated chemistry coupled with LES simulations are a compromise between accuracy and computational time, using the addition of scalar transport equations to predict NO and unburned fuel emissions. The fluid-dynamic fields derived from high-fidelity CFD simulations can then be used in a CFD–CRN approach, which is significantly less computationally intensive compared to the pretabulated and species transport approaches and can efficiently be used to perform sensitivity studies.

The predictive capability of CFD models is found to vary significantly based on the combustion regime, as evidenced by the state-of-the-art analysis. This disparity can be attributed to several factors. First, the chemical kinetics model is found to be a significant contributor to this discrepancy, as there is currently no single model that is valid for all operating conditions.^170^

In the case of perfectly premixed flames, where the chemistry is controlled (apart from preferential diffusion effects), the models are generally easier to solve, and the existing models give satisfactory results and a higher probability of capturing NO_*X*_ emissions, especially for very lean (ϕ ≪1) or very rich (ϕ ≫1) combustion. For ϕ ≫1, the NO_*X*_ chemistry is overconstrained due to the lack of O_2_, allowing good emission prediction; for ϕ ≪1, there are fewer advantages from a chemical/modeling perspective. In addition, the mesh can be more easily calibrated to resolve the flame front (species transport models) or to minimize the error in modeling subgrid effects (pretabulated chemistry models).

However, in multiregime combustion (stratified or nonpremixed flames), where combustion is controlled by mixing/diffusion as well as chemistry, flame thickness resolution becomes a significant challenge for the accurate prediction of NO_*X*_ formation, with a lower probability of capturing NO_*X*_ emissions compared to premixed cases. This problem is further enhanced in lean conditions, where flame thickness is minimal. Conversely, in rich diffusion flames, the flame fronts are significantly thicker, making the prediction of NO_*X*_ formation more feasible and the chemistry more constrained. From a combustion model perspective, it is more difficult to accurately describe the flame front using species transport due to its smaller size (especially in the diffusive regime). In general, very fine meshes must be used to capture the mixing. On the other hand, pretabulated chemistry models have another disadvantage: they can hardly account for multiregime combustion.

## 6 Machine Learning for NO_*X*_ Prediction

Machine learning (ML) has emerged as a promising tool for predicting NO_*X*_ emissions, offering an alternative to traditional experimental methods and computational fluid dynamics (CFD) simulations, provided there are sufficient high-quality data. Its application spans across various combustion systems, including boilers,^247^ thermal power plants,^248^ gas turbines,^249^ and engines,^250^ predominantly focusing on fuels such as coal,^247,248^ natural gas,^249^ diesel,^250^ and biomass.^251^ However, its utilization for the NO_*X*_ prediction of NH_3_ combustion remains limited.

In one of the early works, Li et al.^251^ utilized images of flame radicals such as OH*, CN*, CH*, and C2* as a training data set to predict NO_*X*_ emissions from biomass combustion. The ML model was chosen to be a deep denoising autoencoder (DDAE) which in essence adds noise to the original images and reconstructs the uncorrupted version. The reconstructed images were then compared to the output from a gas analyzer to measure the error. It was reported that the root-mean-square error obtained from the NO_*X*_ predictions were as low as 2 ppm. Ye et al.^252^ used historical operational data in conjunction with data generated from CFD simulations to predict NO_*X*_ emissions in a coal-fired power plant. Three ML models, namely, artificial neural network (ANN), gradient boosted regression trees (GBRT), and support vector regression (SVR), were compared to find the best suitable framework. Through a Shapley additive interpretation analysis, the most important features playing a role in NO_*X*_ production were identified. Finally, the correlation coefficient (*R*^2^) of the model was reported to be 0.98. Yuan et al.^248^ applied an ensemble ML method to predict NO_*X*_ emissions in a coal-fired power plant. Initially, the input feature space consisted of 31 auxiliary variables and then was reduced to 12 variables using principal component analysis and mutual information. Then, three base learners, namely, ANN, decision trees (DT), and SVR were used to create a linear regression based meta-model for NO_*X*_ prediction. The reported *R*^2^ values ranged between 0.87 and 0.95, showing good prediction accuracy. Yan et al.^253^ predicted the NO_*X*_ emissions from a coal bed methane fueled rich-quench-lean combustor using XGBoost, NGboost, and random forests. The model used only four parameters: air mass flow rate, air inlet temperature, swirler angle, and lean burn zone length of the combustor. It was reported that the inlet air temperature had the highest importance in NO_*X*_ production while the swirler angle had no impact on NO_*X*_ emissions. Later, Zhang et al.^254^ applied least-squares SVR (MLS-SVR) to predict CO_2_, CO, and NO_*X*_ emissions from a biodiesel–H_2_ fueled compression ignition engine. The input features for the model were chosen to be thermophysical parameters of the fuel (calorific value, cetane number, density, and viscosity), engine variables (speed and load), and fuel H_2_ content. The model was then used to analyze NO_*X*_ emissions at different engine speeds and loads using the ML model. Overall, the prediction accuracy of the model was satisfactory (*R*^2^ > 0.99). Even though successful applications of ML to the NO_*X*_ prediction of various fuels and combustion systems are present in the literature, applications to NH_3_ combustion systems remain limited.

In a relatively recent first attempt, Saleem et al.^255^ leveraged published literature data to develop artificial neural network (ANN) models for predicting NO_*X*_ emissions from a range of fuels including NH_3_, H_2_, natural gas, kerosene, and their binary blends. It was noted that the NO_*X*_ emissions were highly sensitive to the equivalence ratios for all fuels. Notably, the developed model is agnostic to geometric variations within combustors, exhibiting robust performance with an achieved coefficient of *R*^2^ higher than 0.95, making it suitable for a wide range of applications. Subsequently, Mao et al.^256^ utilized ANNs to predict NO_*X*_ emissions from NH_3_/H_2_ combustion across a wide range of operating conditions. Their approach employed training data derived from a chemical reactor network (CRN) model, incorporating perfectly stirred reactors (PSRs), partially stirred reactors (PaSRs), and plug flow reactors (PFRs) to emulate a rich-quench-lean (RQL) two-stage combustion system. Among the tested ANN models, the backpropagation-ANN approach yielded the highest accuracy (*R*^2^ = 0.998), effectively supplanting the CRN network for NO_*X*_ emission predictions. Very recently, Xing and Jiang^257^ developed neural network potentials (NNPs) to investigate NO_*X*_ and N_2_O formation during NH_3_ and NH_3_/H_2_ combustion. The NNPs were used to replace computationally costly reactive molecular dynamics (RMD) simulations. It was reported that the addition of H_2_ to NH_3_ increased the NO and NO_2_ emissions in NH_3_/H_2_ blends while N_2_O was reduced.

The use of NH_3_ as an alternative fuel in compression ignition engines has gained significant attention due to its potential to reduce carbon emissions. However, the challenges associated with NH_3_ combustion, particularly NO_*X*_ emissions, necessitate advanced optimization and control strategies. Recent studies have explored the application of ML to predict and optimize the NH_3_ combustion performance and emissions in compression ignition engines. Data generation typically involves modified single-cylinder or multicylinder engines operated under various conditions. For instance, Elumalai et al. utilized a single-cylinder common rail engine running on NH_3_–biodiesel dual fuel at 80% load and 1500 rpm.^258^ Similarly, Mi et al. investigated an NH_3_–diesel dual-fuel engine across different NH_3_ energy fractions.^259^ These studies systematically vary parameters such as injection timing, pressure, and fuel composition while measuring performance metrics and emissions by using advanced instrumentation.

Several ML approaches have been employed to model and predict engine performance and emissions. Response surface methodology (RSM) is commonly used for developing regression models and identifying optimal operating conditions. Artificial neural networks (ANNs) have gained popularity due to their ability to capture complex nonlinear relationships in engine data. Elumalai et al. compared RSM and ANN models, finding that the ANN approach produced more accurate predictions across various engine responses.^258^ It was reported that the ANN model predicted all engine responses with *R*^2^ > 0.99, demonstrating excellent reproducibility of experimental data.^258^ When validated against experimental results, these models typically show error rates in the range of 1–5%, which is considered acceptable for engine applications. These models are also used for multiobjective optimization to find the best trade-offs between performance and emissions. Elumalai et al.^258^ used RSM combined with a desirability function approach to optimize split injection parameters, achieving a 12.33% increase in brake thermal efficiency while reducing most emissions, though NO_*X*_ emissions increased by 15.62%.

Future research directions include developing more comprehensive models that account for transient engine operation, as most current studies focus on steady-state conditions. In a recent study, Arivalagan et al. demonstrated the potential of long short-term memory (LSTM) networks for capturing temporal dependencies in engine performance data.^260^ They investigated the emissions from a compression ignition engine using NH_3_–diesel fuel blends. The trained LSTM network successfully captured the NO_*X*_ emissions depending on the fuel blend, engine speed, and crank angles.

The utilization of ML presents an opportunity to expedite the design iteration processes significantly compared to conventional experimental and CFD approaches. Despite notable achievements in other fuel types, the studies focusing on ML applications for NH_3_-fueled combustion systems remain scarce. Nevertheless, the reported predictive accuracies of the models in recent studies underscore the potential of ML in NH_3_ combustion for NO_*X*_ prediction.

## 7 Challenges and Perspectives

Ammonia, as a renewable and clean fuel, holds significant potential for industrial applications. However, the challenges associated with NO_*X*_ emissions from ammonia combustion require further research and development. This review provides a comprehensive analysis of the combustion characteristics, NO_*X*_ emission trends, and underlying mechanisms, contributing to a better understanding of the roles of ammonia in sustainable energy and clean fuels.

Despite extensive research, several gaps hinder the broad implementation of ammonia as a fuel. Current kinetic models, while improved, still show discrepancies in predicting NO_*X*_ emissions across various combustion conditions. More accurate models are needed to capture the complex chemistry of ammonia combustion. There is also a need for more comprehensive experimental data, especially under conditions that mimic real-world applications, to validate and refine the computational models. The development of advanced diagnostic tools to measure key species and intermediates in ammonia flames can provide deeper insights into combustion processes. Looking ahead, pivotal research directions include developing novel strategies for NO_*X*_ reduction, improving CFD models by integrating detailed chemistry and turbulence interactions, and expanding experimental research to cover a broader range of operational parameters. Employing cutting-edge experimental techniques and hybrid experimental–computational approaches can create a synergistic methodology, leveraging the strengths of both to advance the field. By addressing these challenges and pursuing the outlined research directions, significant strides can be made toward the sustainable and efficient use of ammonia as a fuel, with minimal environmental impact.

The field of plasma-assisted NH_3_ combustion presents both significant challenges and promising opportunities, with advancements in several key areas likely to drive the field forward. Future research should focus on developing detailed models that can accurately predict the effects of plasma on NH_3_ combustion dynamics and emission formation under diverse conditions. Advances in computational techniques have the potential to accelerate the generation of critical data for reaction mechanisms, although experimental validation remains challenging to ensure model accuracy. Another important research direction involves exploring novel plasma configurations and discharge types to further enhance combustion performance and emission control. The integration of advanced diagnostics and real-time control systems for plasma-assisted combustion also represents a crucial area for future development. Advancements in plasma-assisted combustion could benefit greatly from collaboration between the high-temperature combustion and low-temperature plasma communities. Furthermore, although current research has primarily focused on NO_*X*_ emissions, future studies should expand to comprehensively address other potential pollutants and their formation mechanisms in plasma-assisted NH_3_ combustion. Additionally, there is a pressing need to investigate the scalability of plasma-assisted-combustion technologies for practical implementation in gas turbines and other large-scale energy systems. This includes addressing challenges related to plasma stability, uniformity, and energy efficiency in high-power applications. Lastly, as the field progresses, there is a need for standardized methodologies and metrics to facilitate meaningful comparisons between different plasma-assisted-combustion strategies and conventional approaches.

While MILD combustion of NH_3_ and its mixtures shows promise for ultralow NO_*X*_ emissions, several challenges and research gaps remain. The necessity for high levels of dilution and a low thermal energy density poses significant design and operational challenges, particularly for practical applications. Future research should focus on optimizing reactor designs to achieve stable MILD combustion conditions while maintaining a high thermal efficiency. There is a need for more comprehensive studies on the effects of pressure, as most current research is limited to atmospheric conditions. The interplay between NH_3_ and various cofuels (e.g., H_2_, CH_4_, syngas, alcohols) in MILD conditions requires further investigation, especially regarding emission control and combustion stability. Advanced diagnostic techniques and high-fidelity numerical simulations are needed to better understand the complex chemical kinetics and flow patterns in NH_3_ MILD combustion. The development of more accurate and comprehensive chemical kinetics mechanisms for NH_3_ and its blends under MILD conditions is crucial. Future work should also explore the scalability of NH_3_ MILD combustion systems from laboratory scale to industrial applications. Additionally, the potential of MILD combustion in mitigating other pollutants, such as N_2_O, deserves attention. Lastly, interdisciplinary research combining combustion science with materials engineering could lead to innovative reactor designs and materials capable of withstanding the unique conditions of NH_3_ MILD combustion.

The modeling of emissions requires further studies and analyses due their complex nature, particularly in instances where H_2_ enrichment is considerable or combustion regimes cannot be approximated with 1D flame structure variations, such as those observed in MILD combustion. The flame morphology can restrict the applicability of models that are commonly used for combustion and emissions predictions. Due to the high complexity of the numerical setup, which incorporates turbulence, radiation, detailed chemistry, and other factors, the precision of emissions predictions is challenging to achieve. Therefore, it is essential to carefully examine the turbulence–chemistry interaction and the impact of supporting models in accordance with experimental data within the turbulent flow field.

In the application of manifold-based reduced chemistry, such as flamelet-generated manifold (FGM) and flamelet/progress variable (FPV), identifying suitable progress variables for predicting NO_*X*_ emissions is critical to progressing with these methods for the analysis of ammonia combustion. Although different methodologies have been proposed in past decades for hydrocarbon fuels, their validity for ammonia systems mixed with different cofuels still needs to be confirmed. Furthermore, it is also desirable for the use of manifold-based reduced chemistry to be invariant with respect to the choice of progress variables, which can be achieved by using reaction–diffusion manifolds (REDIMs).

The modeling and calculation of NO_*X*_ emissions in ammonia combustion through CFD is an important requirement for fundamental and practical use due to the complex interaction between chemical kinetics and turbulent flows, which requires a high level of accuracy and precision. The accurate prediction of NO_*X*_ necessitates the utilization of comprehensive chemical mechanisms that accurately reflect the slower fuel-bound NO_*X*_ formation pathways, particularly in industrial geometries where the computational costs associated with high-fidelity models such as LES render them unreasonable. Although species transport models offer high accuracy, their computational demands render them unfeasible for large-scale applications. Pretabulated models provide a more computationally efficient alternative; however, they are unable to accurately capture the distinct NO_*X*_ formation time scales. The potential of emerging hybrid approaches, such as CRN, to achieve a balance between accuracy and efficiency is significant; however, their success is contingent on the ability of the underlying CFD simulation to replicate the essential combustion characteristics. The perspectives include the refinement of these models to enhance NO_*X*_ prediction accuracy, particularly in lean ammonia–hydrogen blends, while continuing to explore the integration of reduced kinetic mechanisms and advanced turbulence–chemistry interaction models to manage computational resources effectively.

The application of ML to NO_*X*_ prediction in NH_3_ combustion systems presents both significant opportunities and challenges. While ML has shown promise in predicting NO_*X*_ emissions for various fuels and combustion systems, its application to NH_3_ combustion remains limited, representing a critical research gap. A key challenge lies in acquiring sufficient high-quality data specific to NH_3_ combustion, which is essential for developing accurate and robust ML models. Future research should focus on generating comprehensive data sets that capture the complex relationships between operating conditions, fuel compositions, and NO_*X*_ emissions in NH_3_-fueled systems. Additionally, there is a need to extend ML applications beyond steady-state conditions to account for transient engine operations, as demonstrated by recent work on LSTM networks. The development of more sophisticated ML architectures that can integrate multiscale and multiphysics aspects of NH_3_ combustion could significantly enhance predictive capabilities. Furthermore, future research directions should explore the combination of ML with traditional CFD and chemical kinetics models to create hybrid approaches that leverage the strengths of both methodologies. This could lead to more accurate and computationally efficient tools for designing and optimizing NH_3_ combustion systems. As the field progresses, it will be crucial to develop standardized benchmarks and validation methodologies to ensure the reliability and transferability of ML models across different NH_3_ combustion applications. Ultimately, addressing these challenges and pursuing these research directions will be vital to realizing the full potential of ML in advancing NH_3_ as a sustainable fuel option while effectively managing NO_*X*_ emissions.

## 8 Conclusions

Ammonia possesses significant potential as a zero-carbon fuel, offering advantages for hydrogen storage and renewable energy delivery. Its viability for production, preservation, and distribution makes ammonia an appealing option for meeting the requirements of future energy systems geared toward a low-carbon economy. However, widespread adoption of ammonia as a fuel is hindered by challenges such as NO_*X*_ emissions and suboptimal combustion performance, including high ignition point, low combustion speed, and temperature limitations under certain conditions.

This paper provides a comprehensive review of the impacts associated with ammonia combustion. It highlights the existing knowledge gaps that necessitate further research and development to address emission reduction challenges related to NO, N_2_O, unburned ammonia, and carbon monoxide during cofiring. The review also examines recent studies on NO_*X*_ emissions from ammonia flames under different conditions, exploring the pathways through which these emissions occur. Moreover, it emphasizes the need for improving efficiencies to enhance the economic viability of the ammonia combustion technology.

Furthermore, the review delves into the extinction limit of turbulent ammonia flames, discussing influential factors and strategies to promote flame stability. The integration of improved reaction mechanisms, computational models, and a deeper understanding of fundamental phenomena and practical implications associated with ammonia usage enriches this review, making it a valuable resource for disseminating the latest research and developments in the field of ammonia combustion.

## Data Availability

No data is associated with this paper.
